# Fibroblast Growth Factor 2‐Mediated Regulation of Neuronal Exosome Release Depends on VAMP3/Cellubrevin in Hippocampal Neurons

**DOI:** 10.1002/advs.201902372

**Published:** 2020-01-28

**Authors:** Rohit Kumar, Qilin Tang, Stephan A. Müller, Pan Gao, Diana Mahlstedt, Sofia Zampagni, Yi Tan, Andreas Klingl, Kai Bötzel, Stefan F. Lichtenthaler, Günter U. Höglinger, Thomas Koeglsperger

**Affiliations:** ^1^ Department of Translational Neurodegeneration German Centre for Neurodegenerative Diseases Feodor‐Lynen‐Str. 17 81377 Munich Germany; ^2^ Department of Neurology Ludwig Maximilian University Marchioninistr. 15 81377 Munich Germany; ^3^ Graduate Program for Experimental Medicine Faculty of Medicine Technical University of Munich Ismaninger Straße 22 81675 München Germany; ^4^ Department of Neuroproteomics German Centre for Neurodegenerative Diseases Feodor‐Lynen‐Str. 17 81377 Munich Germany; ^5^ Plant Development and Electron Microscopy Department of Biology I Biocenter Ludwig Maximilian University Großhaderner Str. 2 82152 Planegg‐Martinsried Germany; ^6^ Neuroproteomics Klinikum rechts der Isar Institute for Advanced Study Technical University of Munich Ismaninger Straße 22 81675 Munich Germany; ^7^ Department of Neurology (OE 7210) Hannover Medical School Carl‐Neuberg‐Str. 1 30625 Hannover Germany; ^8^ Department of Neurology Technical University of Munich Ismaninger Str. 22 81675 Munich Germany

**Keywords:** CD63, exosome release, fibroblast growth factor, multivesicular bodies, pHluorin, VAMP3

## Abstract

Extracellular vesicles (EVs) are endogenous membrane‐derived vesicles that shuttle bioactive molecules between glia and neurons, thereby promoting neuronal survival and plasticity in the central nervous system (CNS) and contributing to neurodegenerative conditions. Although EVs hold great potential as CNS theranostic nanocarriers, the specific molecular factors that regulate neuronal EV uptake and release are currently unknown. A combination of patch‐clamp electrophysiology and pH‐sensitive dye imaging is used to examine stimulus‐evoked EV release in individual neurons in real time. Whereas spontaneous electrical activity and the application of a high‐frequency stimulus induce a slow and prolonged fusion of multivesicular bodies (MVBs) with the plasma membrane (PM) in a subset of cells, the neurotrophic factor basic fibroblast growth factor (bFGF) greatly increases the rate of stimulus‐evoked MVB‐PM fusion events and, consequently, the abundance of EVs in the culture medium. Proteomic analysis of neuronal EVs demonstrates bFGF increases the abundance of the v‐SNARE vesicle‐associated membrane protein 3 (VAMP3, cellubrevin) on EVs. Conversely, knocking‐down VAMP3 in cultured neurons attenuates the effect of bFGF on EV release. The results determine the temporal characteristics of MVB‐PM fusion in hippocampal neurons and reveal a new function for bFGF signaling in controlling neuronal EV release.

## Graphical abstract

Extracellular vesicles (EVs) are endogenous membrane‐derived vesicles that shuttle biomolecules from cell to cell. The molecular mechanisms that modulate EV release in the central nervous system are poorly understood. Here, it is demonstrated that fibroblast growth factor 2 increases the expression of VAMP3/cellubrevin to enhance the release of EVs from hippocampal neurons.

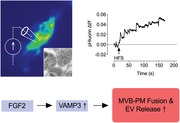

## 1 Introduction

Extracellular vesicles (EVs) are small membrane‐derived vesicles that shuttle bioactive macromolecules such as cytosolic and membrane proteins, RNA, or DNA between cells.^1^ EVs travel wide distances in bodily fluids and are well suited to act as long‐range messengers that deliver state‐dependent molecular information within (and across) organs. Moreover, EVs‐based approaches hold great potential for their use as diagnostic or therapeutic nanocarriers (for review see refs. 2, 3). As determined by their biogenesis, at least three main subgroups of EVs have been defined: ectosomes (also termed microvesicles), exosomes, and apoptotic bodies. These entities originate from different cellular sites: microvesicles (50–1000 nm) are budding directly from the plasma membrane (PM), whereas exosomes (40–150 nm) are derived from multi‐vesicular bodies (MVBs) of the late endo‐lysosomal pathway, where exosomes are formed by inward budding of the limiting membrane into the MVB lumen. The biogenesis of EVs is either assisted by the endosomal sorting complex required for transport (ESCRT), or alternatively, is driven by cone‐shaped lipids, such as ceramide.^4^ The same cell can state‐dependently release different subpopulations of exosomes and exosome‐like EVs with diverse cargo compositions to induce various effects on target cells.^5^, ^6^, ^7^

Before exosomes can be released into the extracellular space, MVBs must fuse with the PM and a number of molecular mechanisms facilitate the otherwise energetically unfavorable MVB‐PM fusion and exosome release. The family of Rab GTPases was shown to control MVB inward budding, transport of MVBs, and MVB‐PM docking in a cell‐type specific manner.^8^, ^9^, ^10^, ^11^, ^12^, ^13^ In addition, MVB‐PM fusion may be facilitated by different soluble NSF‐attachment receptor (SNARE) protein family members in different model systems.^12^, ^14^, ^15^, ^16^, ^17^, ^18^, ^19^, ^20^ Similar to canonical neurotransmission, EV‐release appears to be calcium‐responsive in most cases,^12^, ^21^, ^22^ although MVB‐PM fusion may also be triggered through alternative calcium‐independent mechanisms.^20^

In the central nervous system (CNS), EVs released by neurons and glial cells contribute to the complex networks of cell‐to‐cell signals that underlie CNS physiology and pathology.^23^, ^24^, ^25^ Emerging evidence indicates that EV‐mediated signaling regulates neuronal firing,^26^, ^27^, ^28^, ^29^ synaptic plasticity,^17^, ^30^, ^31^, ^32^, ^33^ and myelin formation.^34^, ^35^ The presence of EVs in cerebrospinal fluid (CSF) underscores their relevance in neural tissue.^36^ In the injured CNS, EVs were recently found to cross the blood–brain barrier and promote neuroinflammation.^37^, ^38^ Furthermore, certain types of EVs appear to support neuronal survival under conditions of ischemic stress.^9^, ^26^, ^39^ In neurodegenerative diseases, EVs contribute to the seeding and spreading of toxic protein aggregates, and influence the aggregation process and clearance of these aggregates.^40^

In EV‐mediated neuron–glia communication, a general theme is that the secretion of EVs appears to be coupled to neuronal activity. Neuronal depolarization and glutamate release has been suggested to stimulate the secretion of exosomes from neurons^41^, ^42^, ^43^, ^44^, ^45^ and oligodendrocytes^9^ while serotonin is reported to stimulate microglial secretion of EVs.^46^ The EVs may in turn transfer bioactive RNAs, proteins, and lipids between cells, thereby regulating neuronal activity.^17^, ^25^, ^30^, ^31^, ^33^, ^41^ Similar to neurotransmitter vesicles fusing with the PM, exosome release can be evoked by electrical activity in conjunction with calcium influx and depends on SNARE proteins. However, the temporal characteristics of stimulus‐evoked EV release and cellular‐molecular mechanisms that segregate it from neurotransmitter release are currently unknown.

Fibroblast growth factors (FGFs) are a family of pleiotropic growth and differentiation factors that regulate CNS homeostasis in health and disease. Although FGFs are best known for their roles in the early steps of patterning of the neural primordium and proliferation of neural progenitors, they have equally important roles in the adult brain; where they regulate neuronal calcium homeostasis and plasticity and promote neuroprotection and repair in response to neural tissue damage (reviewed in ref. 47). In addition to these physiological roles, bFGF has been implicated in responses to neuronal injury^48^, ^49^, ^50^ or psychiatric conditions (reviewed in refs. 51, 52). For instance, a large body of evidence implicated FGF2 and the FGFR in mood and anxiety disorders (reviewed in refs. 51, 52) where FGF2 has been consistently found to be decreased in post‐mortem brain tissue from depressive subjects and in the brain of animal models including the hippocampus,^53^, ^54^, ^55^, ^56^, ^57^ which is a highly relevant region for mood disorders.^58^ Single nucleotide polymorphism (SNP) in the FGF2 gene was found to be associated with side effects and responsiveness to antidepressant treatment.^59^ Conversely, chronic antidepressant or anxiolytic treatment resulted in an increase in FGF2^60^ and administration of FGF2 exerted antidepressant properties.^61^, ^62^ FGFs are expressed throughout the developing and adult CNS, with FGF‐1 and FGF‐2 (basic FGF or bFGF) being the most abundant FGF family member in the hippocampus.^63^, ^64^, ^65^, ^66^ bFGF is released from glia and—to a lesser degree—from hippocampal neurons^67^ and exhibits the highest affinity for the FGF receptor 1 (FGFR1).^68^ Conversely, FGFR1 is the most abundant FGF receptor subtype in the hippocampus, where it is predominantly expressed in CA1/CA3 pyramidal neurons^69^, ^70^, ^71^, ^72^ (reviewed in ref. 65). Despite their important role in hippocampal homeostasis, the contribution of FGFs to regulating neuronal EV release has not yet been addressed.

Here, we used a combination of patch‐clamp electrophysiology and pH‐sensitive dye imaging to examine stimulus‐evoked EV release in individual neurons in real time. We found that cultured hippocampal neurons have a low consecutive rate of spontaneous EV release events. Different from previous reports,^41^, ^42^, ^43^, ^44^, ^45^ we found that high‐frequency electrical stimulation (HFS) does not reliably induce EV release in cultured hippocampal neurons and that only a small subset of cells responds to HFS with a burst‐like increase of MVB‐PM fusion events. Unlike synaptic vesicles, which fuse with the PM within milliseconds in response to PM depolarization, the stimulus‐evoked fusion of MVBs occurred only after tens of seconds and exhibited a significant time lag between the initial stimulus‐evoked calcium signal and the eventual MVB‐PM fusion event. Treatment with basic fibroblast growth factor (bFGF) greatly increased the rate of stimulus‐evoked MVB‐PM fusion events and consequently the abundance of EVs in the culture medium. Further proteomic analysis of neuronal EVs demonstrated bFGF to increase the abundance of the v‐SNARE vesicle‐associated membrane protein 3 (VAMP3, cellubrevin) on EVs. Conversely, knocking‐down VAMP3 in cultured neurons attenuated the effect of bFGF on EV release. In summary, our results thus describe for the first time the specific temporal characteristics of MVB‐PM fusion in hippocampal neurons in response to electrical stimulation, uncovering a significant temporal separation of neuronal calcium influx and MVB‐PM fusion. In addition, our data reveal a new function of bFGF signaling in controlling neuronal EV release and thus support the investigation of growth factor‐mediated signal transduction via EVs in the healthy and diseased CNS.

## 2 Results

### 2.1 Stimulus‐Induced MVB‐PM Fusion Has an Abate Success Rate and Slow Temporal Kinetic

A number of reports connected EVs release to neuronal activity in conjunction with calcium influx.^41^, ^42^, ^43^, ^44^, ^45^ However, because these studies examined bulk release in EV‐enriched medium pellets, the specific spatial and temporal kinetics of neuronal MVBs fusion in response to a depolarizing stimulus are currently unknown. In order to visualize MVB‐PM fusion in individual cells, we transduced primary hippocampal neurons after 3 days in vitro (DIV) with a lentivirus to express pCD63‐pHluorin^20^, ^22^, ^73^, ^74^ (Figure S1a, Supporting Information). Compared to the neutral pH value of the extracellular compartment (7.4), the endosomal and lysosomal lumen pH is maintained in a range of 4.5–6.5, due to the activity of ATP‐dependent proton pumps in the membrane of endosomes.^75^ Because of the topological orientation of the tag, with pHluorin facing the acidic intraluminal side of the MVB, the fluorescence emitted from pHluorin is quenched. Upon fusion of the MVB with the PM, the low luminal pH is immediately neutralized, resulting in an increase in fluorescence intensity. Different from examining bulk release in EV‐enriched medium pellets, this approach therefore allows for the visualization of MVB‐PM fusion events in single cells.

**Figure 1:**
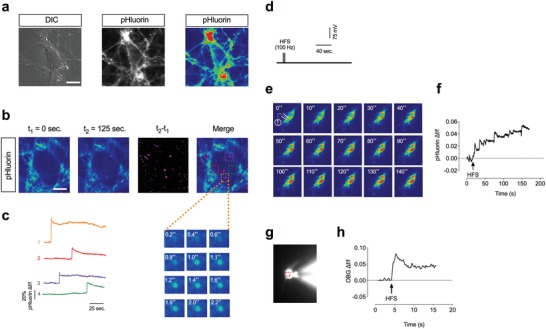
High‐frequency stimulation (HFS) evokes MVB‐PM fusion in a subset of neurons. a) Photomicrograph illustrating a cultured rat hippocampal neuron (DIV 12) transduced with *p*CD63‐pHlurin under differential interference contrast (DIC) (left). Photomicrograph of the same cell with the focus plane adjusted to the the plasma membrane and under fluorescence microscopy (excitation at 488 nm) in b/w (middle) and in pseudo color (right). b) Representative photomicrographs from time lapse fluorescence live‐cell imaging (excitation wave length at 488 nm) from a single cell at *t*_1_ = 0 s and *t*_2_ = 125 s in pseudo color (first and second from left). Subtracting both images from each other (*t*_2_ − *t*_1_) represents the sum of changes in fluorescence over time. Changes in fluorescence in the last as compared to the first frame are indicated in purple (third from left). Projection image with the images from (d) and (f) merged (fourth from left). c) Representative fluorescence traces (Δf/f) illustrating the rise in fluorescence in the ROIs indicated in (b). The respective ROIs are color‐coded in red, purple, green, and orange. High magnification microscopic image illustrating a burst‐like increase in fluorescence in the first ROI (orange). d) Schematic illustrating the high‐frequency stimulus (HFS) protocol applied in cultured hippocampal neurons. A HFS has been given for 1 s at 100 Hz. e) Representative images in pseudo color from live‐cell imaging of patch‐clamped CD63‐pHlurin‐transduced hippocampal neurons during and after HFS. The CD63‐pHlurin fluorescence signal remained stable as long as the holding membrane potential was maintained at *V*_Hold_ (−70 mV) prior to the HFS. A burst increase in fluorescence is elicited in response to a HFS. f) Representative fluorescence trace illustrating a burst‐like increase in CD63‐pHlurin fluorescence following HFS in the cell indicated in (d). g) Photomicrograph illustrating a hippocampal neuron filled with Oregon Green BAPTA 488 through the patch pipette. h) Graph illustrating a rise in cytosolic calcium in response to HFS from the ROI in red in (g).

At day 12 post‐transduction with pCD63‐pHluorin, CD63‐pHluorin fluorescence co‐localized with Rab7‐positive late endosomal vesicles (Figure S1b,c, Supporting Information) and strongly increased upon perfusion with 50 mm NH_4_Cl (Figure S1d,e, Supporting Information), thus confirming a pH‐dependent quenching of fluorescence in acidic cell organelles. We examined the cell culture medium from CD63‐pHluorin‐transduced neurons and found GFP to be present in EV‐enriched medium pellets together with the EV proteins CD81 and Alix/AIP1 (Figure S1f, Supporting Information). When loaded on iodixanol gradients, CD81 and Alix/AIP1 segregated to low‐density fractions, consistent with the typical density of exosomes^5^ (Figure S1f, Supporting Information). Compared to CD81 and Alix/AIP1, the distribution of GFP on the gradient was found to be somewhat broader, indicating that CD63‐pHluorin labels a more heterogeneous population of EVs (Figure S1f–i, Supporting Information). Based on these results we concluded that CD63‐pHluorin is successfully distributed to MVBs/late endosomes and to exosome‐like EVs in primary hippocampal neurons. Live cell imaging of CD63‐pHluorin‐expressing neurons (**Figure** **1**a,b) over a period of 5 min revealed sudden increases in fluorescence, suggestive of MVB‐fusion events in a small number of cells (*n* = 4/34 cells, 11.7%) (Movie S1, Supporting Information). Each event consists of a punctate, burst‐like increase in fluorescence with a comparably long signal duration and slow decay rate (Figure 1c) and occurred over a period of several minutes (avg. event rate: *n* = 2 ± 0.65 events per cell per minute). MVB‐PM fusion events were predominantly located to the soma, whereas axo‐dendritic EV release was extremely scarce in cultured neurons. We concluded that cultured hippocampal neurons have a low consecutive MVB‐PM rate under unstimulated conditions.

When whole‐cell patch‐clamped, a subset of CD63‐pHluorin‐transduced neurons (*n* = 5/34 neurons, 14.7%) likewise exhibited a series of rapid fluorescence bursts in response to a brief high‐frequency stimulus (HFS) of 100 Hz over 1 s (Figure 1d; Movie S2a,b, Supporting Information). In HFS‐responsive cells, the burst‐like increase in pHluorin‐fluorescence occurred on average after an interval of 38.51 s (38.51 ± 12.54 s) between the HFS and the first burst with a relatively slow temporal rate ranging from seconds to minutes (*t*_1/2_ = 86.83 ± 0.6149 s; *n* = 5) and no immediate gross decay or signal termination was observed during recording (Figure 1e,f). Conversely, the same HFS evoked a fast and immediate increase in intracellular calcium as measured by the calcium indicator Oregon Green 488 BAPTA‐1 (Figure 1g,h). These results thus demonstrate that stimulus‐evoked MVB‐PM fusion has an abated success rate in cultured neurons and a comparably long time lag between the stimulus and MVB‐PM fusion. In order to further investigate the role of intracellular calcium stores for EV release, we perfused cultured neurons with thapsigargin (10 µm) while imaging CD63‐phluorin fluorescence (Figure S2, Supporting Information). Thapsigargin is a non‐competitive inhibitor of the sarco/endoplasmic reticulum Ca^2+^ ATPase (SERCA) that raises cytosolic (intracellular) calcium concentrations by blocking the ability of the cell to pump calcium into the sarcoplasmic and endoplasmic reticula.^76^ Whereas we detected EV release events in response to HFS, perfusion of cultured neurons with thapsigargin had no effect (*n* = 60 neurons) (Figure S2c, Supporting Information).

### 2.2 Basic Fibroblast Growth Factor Increases Stimulus‐Evoked MVB‐PM Fusion

**Figure 2:**
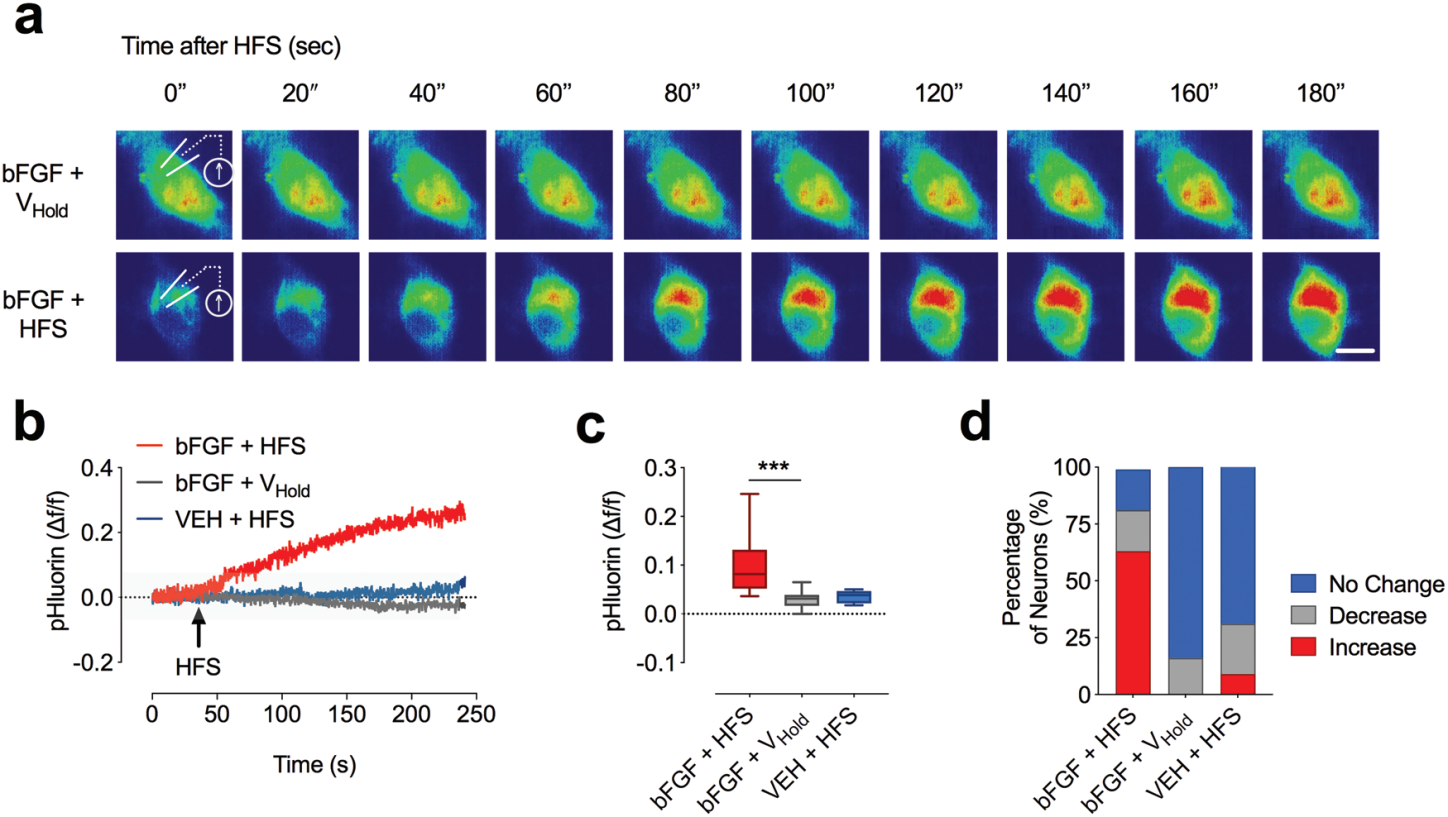
bFGF increases stimulus‐induced CD63‐pHluorin fluorescence indicative of MVB‐PM fusion. a) Images in pseudo color illustrating an increased fluorescence signal in bFGF‐treated neurons in response to a HFS. b) Representative fluorescence traces illustrating an increase in CD63‐pHluorin fluorescence following HFS in VEH‐ and bFGF‐treated neurons and in unstimulated cells (*V*_Hold_). c) Bar graph illustrating the average maximal fluorescence in response to a HFS (number of cells/condition: bFGF + HFS vs bFGF + *V*_Hold_; *n* = 17 and 10; *p* = 0.0007). d) Bar graph demonstrating the relative proportion of neurons that exhibited an increase or decrease of fluorescence or no change in response to a HFS in the same groups (number of cells/condition (bFGF + HFS; *n* (increase) = 31, *n* (decrease) = 9, *n* (no change) = 9 (*n* total = 49); bFGF + *V*_Hold_; *n* = *n* (increase) = 2, *n* (decrease) = 1, *n* (no change) = 7 (*n* total = 10); VEH + HFS; *n* = *n* (increase) = 1, *n* (decrease) = 1, *n* (no change) = 9 (*n* total = 11), respectively). Data are shown as mean ± s.e.m. For comparison, a two‐tailed unpaired *t*‐test was used in (c). ***p < 0.001.

We next aimed to identify candidate molecules that regulate MVB‐PM fusion in hippocampal neurons. Among other candidates, we investigated the effect of bFGF on MVB‐PM fusion. When we treated cultured hippocampal neurons after 9–12 days in vitro (DIV) for 24 h with bFGF (50 ng mL^−1^), we found the proportion of neurons exhibiting a HFS‐evoked increase in CD63‐pHluorin fluorescence to be greatly enhanced (**Figure** **2**a; Movie S3a–d, Supporting Information). In bFGF‐treated cells, CD63‐pHluorin fluorescence increased over a period of several minutes (*t*_1/2_ = 117.8 ± 2.67 s; *n* = 17) following a HFS (Figure 2b,c). Overall, the proportion of neurons that exhibited an increase in fluorescence increased to ≈60% in bFGF‐treated and HFS‐stimulated neurons (Figure 2d).

**Figure 3:**
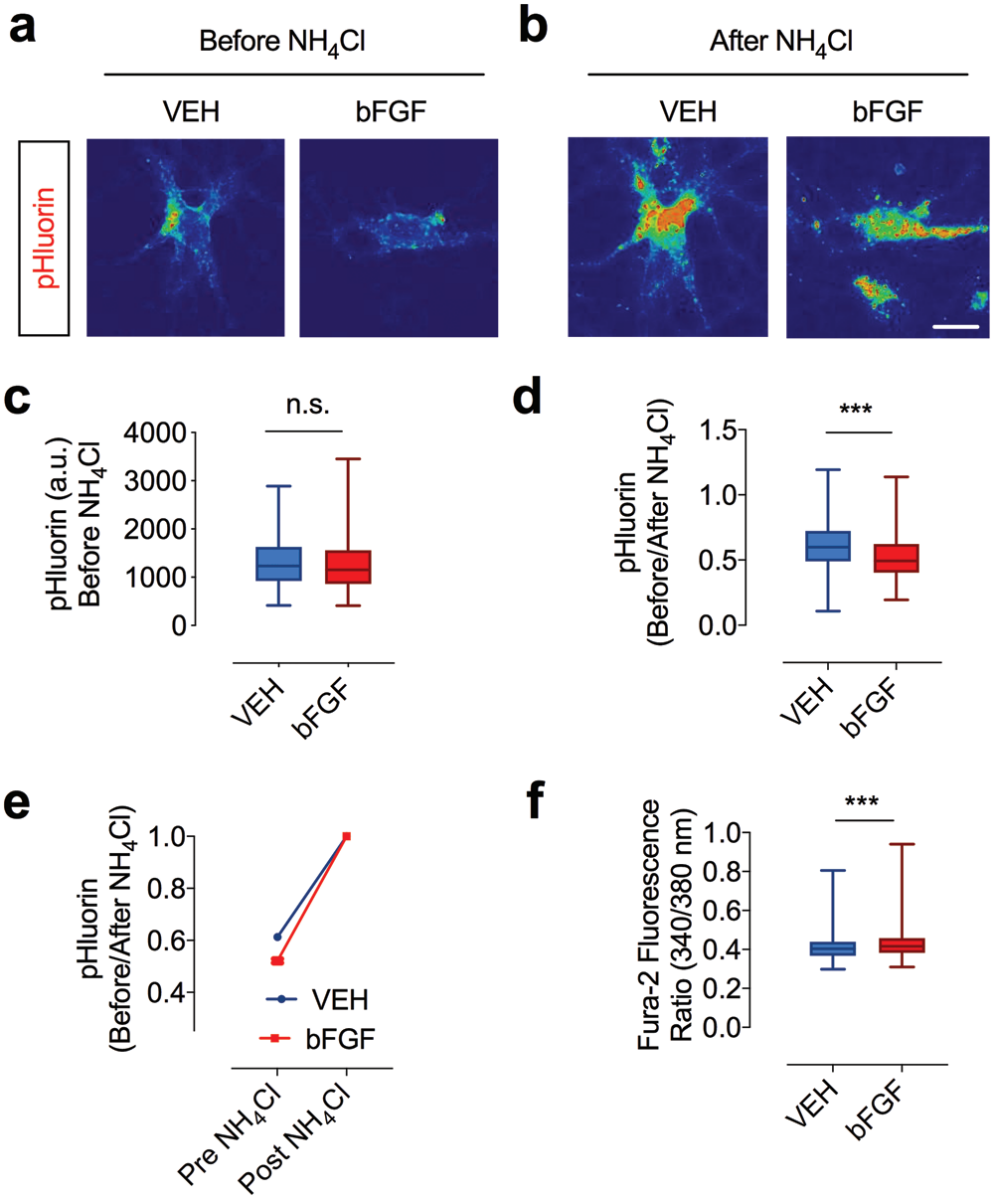
bFGF treatment results in acidification of CD63‐positive multivesicular endosomes. a,b) Photomicrographs illustrating CD63‐pHluorin fluorescence in unstimulated bFGF‐ and VEH‐treated neurons before and after application of NH_4_Cl. c) The raw fluorescence values are similar in bFGF‐ and VEH‐treated neurons (number of cells/condition *n* = 510 and 505; *p* = 0.1488). d,e) Relative to the maximal fluorescence evoked by NH_4_Cl, the CD63‐pHluorin signal in bFGF‐treated neurons (50 ng mL^−1^, 24 h) is slightly decreased, indicating a decreased pH‐value in MVBs of bFGF‐treated neurons (*p* < 0.0001). f) Treatment with bFGF leads to a small increase of the intracellular calcium concentration as measured by Fura 2 (number of cells/condition *n* = 312; *p* = 0.0003). Data are shown as mean ± s.e.m. For comparison, a two‐tailed unpaired *t*‐test was used. ***p < 0.001.

In principle, an increase in fluorescence is due to MVB‐PM fusion and exposure of the pHluorin tag to the extracellular environment. Alternatively, it may be a consequence of an increased pH‐value inside the MVB or an increase in the expression of CD63‐pHluorin. To address the effect of bFGF on the pH‐value inside MVBs, we treated cultured neurons with bFGF and imaged CD63‐pHluorin fluorescence under steady state and after treatment with 50 mm NH_4_Cl (**Figure** **3**a,b). Whereas the absolute fluorescence value was similar in either condition (Figure 3c), we found that bFGF‐treatment (50 ng mL^−1^ for 24 h) led to a significant decrease in fluorescence relative to the maximal NH_4_Cl‐evoked fluorescence signal (Figure 3d,e). These results indicate a reduced pH value inside MVBs in bFGF‐treated neurons. In line with previous reports,^77^, ^78^, ^79^ bFGF treatment also led to an increase in the intracellular calcium concentration as measured by Fura‐2 calcium imaging (Figure 3f). bFGF had no statistical significant effect on the expression of pHluorin over a period of 48 h, although there was a trend toward a reduced pHluorin expression in bFGF‐treated neurons (Figure S3a–c, Supporting Information). Based on our findings, we concluded that the HFS‐evoked increase in CD63‐pHluorin fluorescence in bFGF‐treated neurons represents MVB‐PM fusion rather than an increased pH‐value inside the MVB or the overall expression of CD63‐pHluorin.

### 2.3 bFGF Increases EV Release in Cultured Hippocampal Neurons

**Figure 4:**
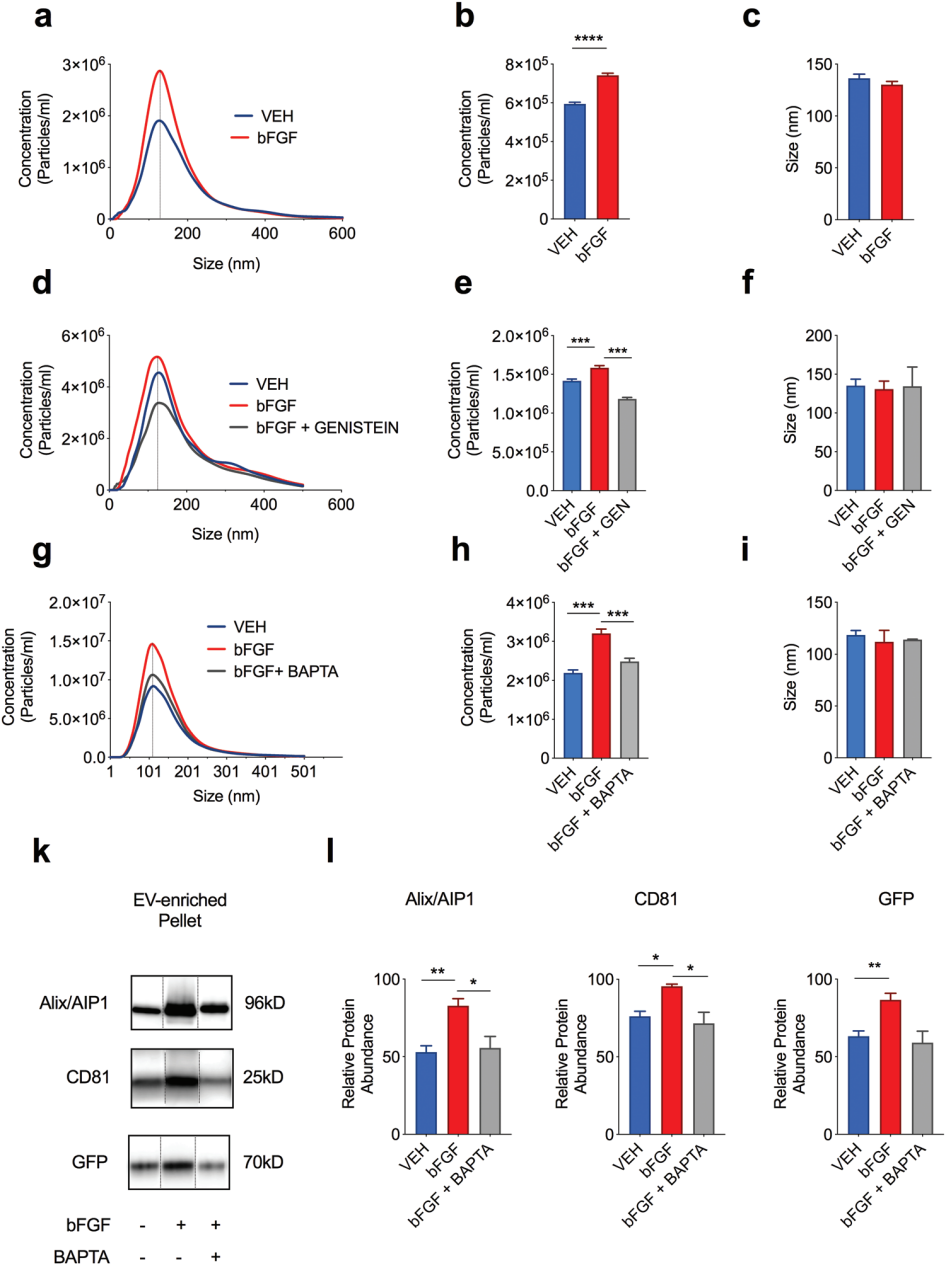
bFGF increases the release of extracellular vesicles (EVs) from cultured hippocampal neurons. a) Averaged curves illustrating the number of particles/size in the cell culture medium of bFGF‐ or vehicle (VEH)‐treated hippocampal neurons as measured by nanoparticle tracking analysis (NTA). b) Bar graphs demonstrating an increased particle number in bFGF‐treated neurons as compared to VEH‐treated cells as measured by NTA (*n* = 11 and 15 samples/condition; *p* < 0.001). c) Treatment with bFGF has no statistical significant effect on the average particle size (*p* = 0.2687). d) Results from NTA illustrating the number of particles/size in medium from bFGF or VEH‐treated hippocampal neurons with or without co‐application of 50 µm genistein (GEN). e,f) Genistein reduces the bFGF‐induced increase in particle number but had no effect on the size of particles (*n* = 5 samples/condition; *p* < 0.001). g) Results from NTA illustrating the number of particles/size in medium from bFGF or VEH‐treated hippocampal neurons with or without co‐application of 1 µm BAPTA‐AM. h,i) BAPTA‐AM normalized the bFGF‐induced increase in particle number but had no effect on the size of particles (*n* = 3 samples/condition; *p* < 0.001). k) Western blot illustrating the abundance of CD81, Alix/AIP1, and GFP in the EV‐enriched pellet of cell culture medium from bFGF and VEH‐treated cells with or without co‐application of 1 µm BABPTA‐AM. l) Bar graphs illustrating an increase of CD81, Alix/AIP1, and GFP in the EV‐enriched pellet of bFGF‐treated neurons (VEH vs bFGF: *n* = 5; *p* = 0.0006 for CD81, *p* = 0.0013 for Alix/AIP1, *p* = 0.0028 for GFP). Co‐application of BABPTA‐AM normalized the bFGF‐induced increase in these proteins in the EV‐enriched pellet. Data are shown as mean ± s.e.m. For comparison, a two‐tailed paired *t*‐test was used in (b) and an unpaired *t*‐test in (e,h,l) ***p < 0.001, **p < 0.01, *p < 0.05.

In order to further examine the effect of bFGF on neuronal EVs, we treated cultured rat hippocampal neurons at DIV 9–12 with bFGF or vehicle (VEH). We quantified the number of EVs in the culture medium of neurons treated with VEH or bFGF by nanoparticle tracking analysis (NTA) and found that bFGF (50 ng mL^−1^ for 24 h) increased the number of EVs without having a significant effect on EV size (**Figure** **4**a–c). Medium EVs had an average size of 130.2 ± 3.15 nm, consistent with the size of exosomes.^1^ Treatment with bFGF (50 ng mL^−1^ for 24 or 48 h) had no statistical significant effect on the release of LDH (Figure S4a, Supporting Information) or the amount of apoptotic neuronal nuclei (Figure S4b,c, Supporting Information), thus demonstrating that the increased number of EVs is not a consequence of neuronal cell death or compromised membrane integrity in bFGF‐treated neurons. FGFs act through FGF receptors that are classified as high‐affinity tyrosine kinase receptors.^80^ In accord, the effect of bFGF on EV number was mediated through activation of tyrosine kinase‐dependent receptors, because co‐application of genistein (50 µm), an inhibitor of (receptor) tyrosine kinase activity, decreased the effect of bFGF to sub‐normal levels (Figure 4d–f). In line with a calcium‐dependent mechanism of bFGF‐induced exosome release, co‐application of the cell‐permeant calcium chelator BAPTA‐AM (1 µm) normalized the effect of long‐term bFGF treatment on exosome release (Figure 4g–i). To further examine the effect of bFGF on exosome release, we quantified the abundance of the exosome‐associated proteins CD81, Alix/AIP1, and GFP (pHluorin) in EV‐enriched medium pellets from bFGF and VEH‐treated neurons. Consistent with our NTA data, we found that treatment with bFGF increased the abundance of these proteins, whereas co‐application of BAPTA‐AM normalized the effect of bFGF on CD81, Alix/AIP1 and GFP (Figure 4k–l). Different from these compounds, transduction of neurons with CD63‐pHluorin alone had no effect on the abundance of EVs (Figure S3d,e, Supporting Information). In summary, these results demonstrate that prolonged (24 h) treatment with bFGF increases neuronal EVs in a calcium‐dependent manner. On the other hand, bFGF had no effect on the size or number of MVBs as measured by immunocytochemistry (ICC) and electron microscopy (EM) (Figure S5, Supporting Information).

### 2.4 bFGF Modulates the Abundance of EV‐Enriched SNARE Proteins

**Figure 5:**
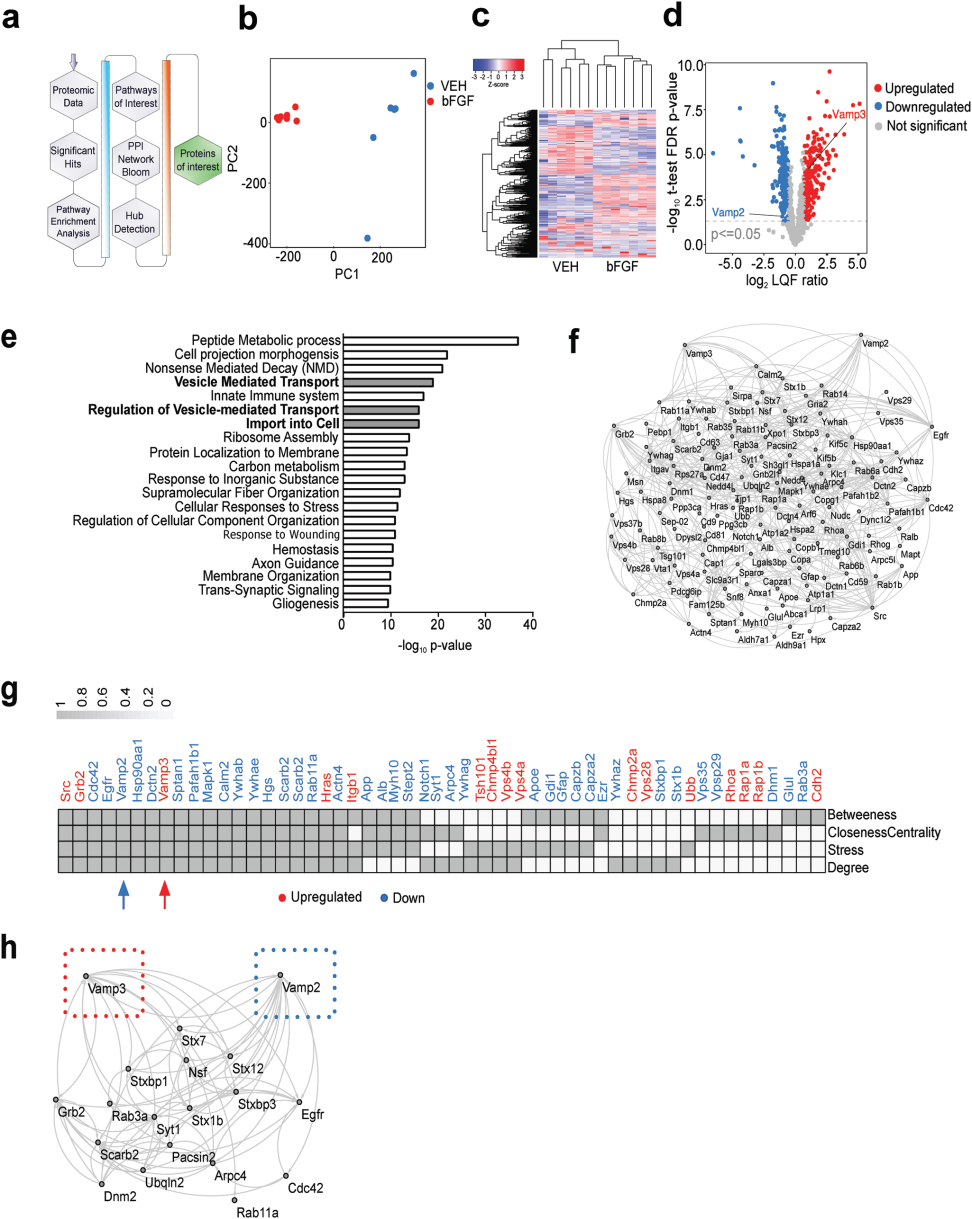
Treatment with bFGF modulates the abundance of SNARE proteins on EVs. a) Schematic illustrating the workflow used to analyse the proteomic data from MS analysis. b) PCA plot illustrating a segregation of the two treatment condition (bFGF vs VEH). c) Hierarchical clustering and heat map showing the relative expression value (*z*‐score and log_2_‐transformed LFQ protein intensities) of 705 differentially abundant proteins (FDR < 0.05) in EVs from bFGF‐ and VEH‐treated neurons. Each column represents a replicate of either treatment condition and each row corresponds to a differentially abundant protein. d) Volcano plot of statistical significance (−log_10_ transformed *p*‐value, −log_10_(0.05) = 1.30) against log_2_ transformed protein LFQ ratios between the two treatment conditions. Blue (decreased) and red (increased) dots indicate proteins with significantly changed abundance. e) Bar graph illustrating the results of a functional pathway analysis based on Gene Ontology, Reactome, and KEGG resources. f) Network analysis illustrating candidate protein interactions. g) Table illustrating the top 53 hub proteins against four centrality calculations in a network topology. h) Protein–protein interaction network illustrating functional interaction partners of VAMP2 and VAMP3. Source to target topology is considered for network depiction.

We instead hypothesized that bFGF affects exosome release through stimulating MVB‐PM fusion. Previous research implicated a number of molecules in mediating MVB‐PM fusion, such as Rab GTPases^8^, ^9^, ^10^, ^11^, ^12^, ^13^ and soluble NSF‐attachment receptor (SNARE) protein family members.^12^, ^14^, ^15^, ^16^, ^17^, ^18^, ^19^, ^20^ The abundance of these molecules on the MVB limiting membrane is likely mirrored by their abundance on exosomes as a result of their mechanism of generation with inward budding into the lumen of MVBs. Investigating exosomal proteins will thus support the understanding of molecular changes of the MVB limiting membrane. Therefore, instead of investigating cell lysates, we performed label‐free quantification (LFQ) of proteins using high‐resolution mass spectrometry (MS) on isolated EV‐enriched pellets from bFGF and VEH‐treated neurons (*n* = 6 per condition) (**Figure** **5**a). Principal component analysis (PCA) revealed a clear discrimination between the treatment conditions (Figure 5b), further confirmed by unsupervised hierarchical clustering of the differentially expressed proteins (Figure 5c). The Pearson's correlation (PC) analysis of log_2_ transformed protein LFQ intensities revealed a clear separation between the two groups and an excellent reproducibility of the replicates within the two groups (Figure S6, Supporting Information). In total 2258 distinctly expressed proteins were identified (Table S1, Supporting Information). In total, 705 proteins showed a significantly changed abundance in EVs (FDR < 0.05) in response to treatment with bFGF. Out of these differentially abundant proteins, 441 proteins had a fold change of 1.5 or above with a *p*‐value < 0.05. Among those 441 proteins, 235 proteins had an increased and 206 proteins had a decreased abundance, respectively (Figure 5d). Based on functional pathway enrichment analysis, we found strong enrichment in the categories “vesicle mediated transport,” “regulation of vesicle mediated transport,” and “import to the cell” (Figure 5e). We next developed an interaction network and ran a centrality analysis to determine critical signaling hub proteins. We computed four network parameters: i) betweenness, ii) closeness, iii) stress, and iv) degree. Based on this analysis, the centrality ranks of 53 proteins were calculated (Figure 5f). Among these proteins, the v‐SNARE proteins vesicle‐associated membrane protein 2 and 3 (VAMP2 and VAMP3) had the highest centrality measures (Figure 5g). Remarkably, bFGF decreased the abundance of VAMP2 (0.69‐fold) whereas it increased the abundance of VAMP3 (1.71‐fold) in EV‐enriched medium pellets. Visualization of their functional interaction partners demonstrated their functional relationship with a variety of proteins implicated in membrane fusion (Figure 5h). In summary, our proteomic data analysis thus demonstrates bFGF to affect key intrinsic molecular mechanisms responsible for vesicle membrane fusion in hippocampal neurons.

### 2.5 bFGF Enhances Neuronal EV Release in a VAMP3 Dependent Mechanism

**Figure 6:**
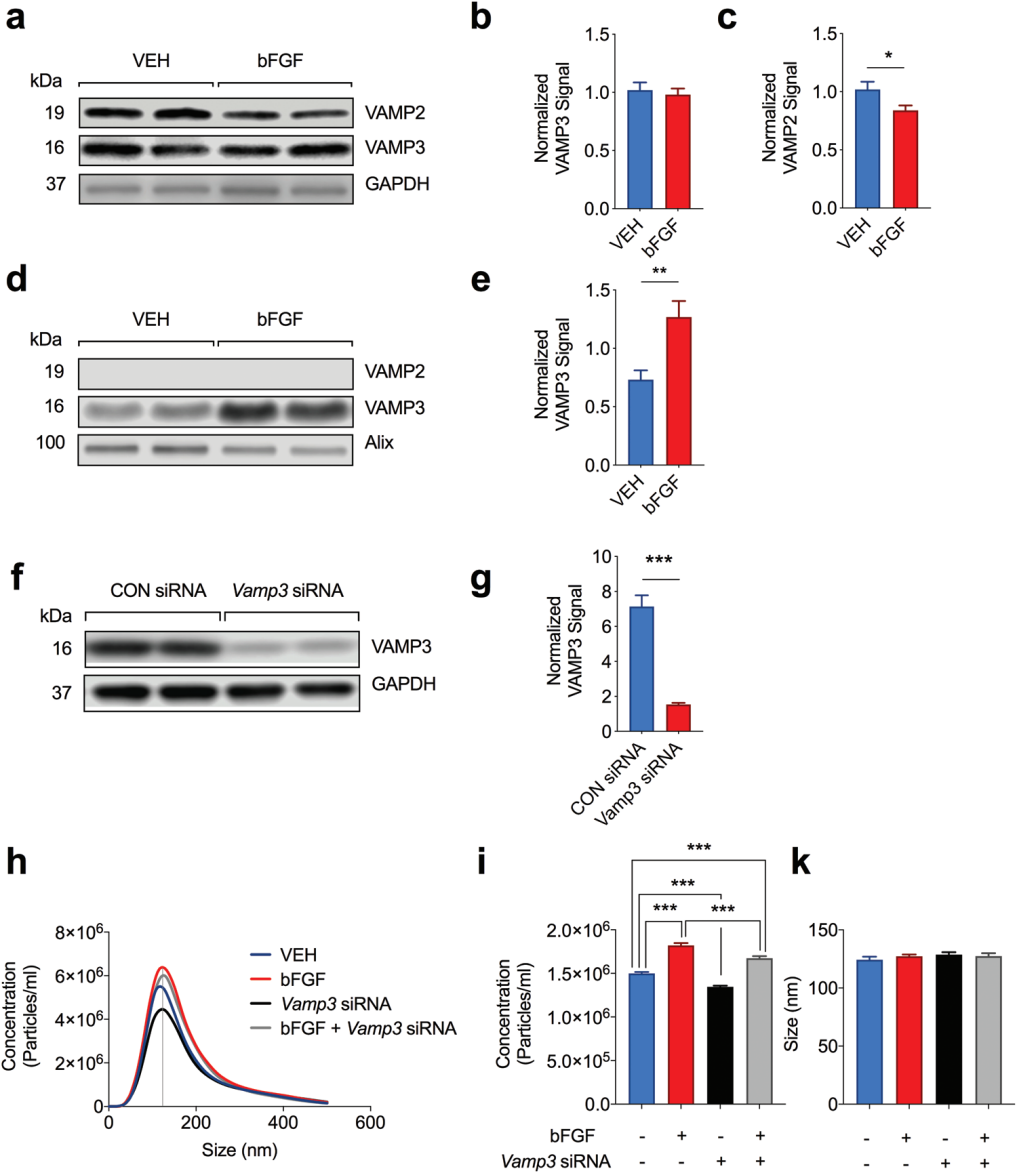
The bFGF‐induced increase in EV release is partly mediated through VAMP3. a) Western blot illustrating the abundance of VAMP2 and VAMP3 in cell lysates from bFGF‐ and VEH‐treated neurons. b,c) The abundance of VAMP2 is decreased in neuronal cell lysates in response to bFGF‐treatment, whereas the abundance of VAMP3 remains unaffected (*n* = 26/condition; VAMP3: *p* = 0.6541; VAMP2: *p* = 0.0252). d) Western blot illustrating the abundance of VAMP2 and VAMP3 in EV‐enriched pellets from bFGF‐ and VEH‐treated neurons. e) Treatment with bFGF increases the abundance of VAMP3 on EVs released from cultured neurons, whereas VAMP2 is undetectable on neuronal EVs (*n* = 12/condition; *p* = 0.0027 f,g) Western blot and bar graph illustrating the knock‐down of VAMP3 in response to treatment with Vamp3 siRNAs (*n* = 12/condition; *p* < 0.001). h) Averaged curves from NTA illustrating the the number of particles/size in the cell culture medium of bFGF or VEH‐treated hippocampal neurons with our without co‐application of Vamp3 siRNAs. i,k) Bar graph illustrating a reduced number of EVs in neurons pre‐treated with Vamp3 siRNA (10 nm for 48 h). Co‐application of Vamp3 siRNAs partially prevents the bFGF‐mediated increase in EVs (VEH vs bFGF vs Vamp3 siRNA vs bFGF + Vamp3 siRNA: number of samples/condition *n* = 24 vs 12 vs 24 vs 12; *p* < 0.0001). Treatment with bFGF or Vamp3 siRNAs has no effect on the size of EVs as measured by NTA. Data are shown as mean ± s.e.m. For comparison, a two‐tailed unpaired *t*‐test was used in (c), (e), and (g) and a paired *t*‐test was used (i) ***p < 0.001, **p < 0.01, *p < 0.05.

Based on our results we hypothesized bFGF to affect MVB‐PM fusion and exosome release by modulating the expression of v‐SNARE proteins. In order to test this hypothesis, we first examined the abundance of VAMP2 and 3 in neuronal cell lysates and EV‐enriched pellets from bFGF‐ or VEH‐treated hippocampal neurons by Western blot. In accord with our MS data, we found the abundance of VAMP2 to be decreased in cell lysates from cultured neurons in response to treatment with bFGF, whereas the abundance of VAMP3 remained unaffected (**Figure** **6**a–c). In contrast to cell lysates, the abundance of VAMP3 was increased in EV‐enriched medium pellets from bFGF‐treated neurons, where VAMP2 was not detectable (Figure 6d,e). Since exosomes are generated from inward budding of MVB membrane patches,^1^ a high level of VAMP3 on exosomes may be reflected by an increased abundance of VAMP3 on the cytoplasmic MVB membrane and thus help to fuse MVBs to the PM. In order to test this hypothesis, we used a siRNAs specific for *Vamp3* to knock‐down VAMP3 in cultured neurons (Figure 6f,g; Figure S7, Supporting Information). Consistent with a role of VAMP3 for EV release, we found that siRNA‐mediated knock‐down of VAMP3 attenuated the number of EVs for both VEH‐ and bFGF‐treated neurons as measured by NTA (Figure 6h,i). In accord with a specific effect of VAMP3 on MVB‐PM fusion, the EV size remained unaffected with Vamp3 siRNA treatment (Figure 6k). Our results therefore suggest that bFGF increases EV release in part through a VAMP3‐dependent mechanism. Our results suggest a non‐redundant functional role of the homologs VAMP2 and VAMP3 for EV release which can serve as a molecular basis for the segregation of MVB and neurotransmitter carrying synaptic vesicle fusion events, requiring the presence of VAMP2.^81^

## 3 Discussion

In our study, we examined the effect of electrical stimulation on neuronal EV release in single cells and in real time. Although cultured hippocampal neurons exhibit a constitutive release of EVs, we found MVB‐PM fusion to be a relatively rare event in cultured neurons under unstimulated conditions (Figure 1). Because the vast majority of our cultured neurons at DIV 9–12 had spontaneous miniature excitatory postsynaptic currents (mEPSCs), spontaneous action potentials (APs), and spontaneous calcium oscillations (data not included), these results suggest that basal neuronal activity is uncoupled from neuronal MVB‐PM fusion and exosome release. Our data therefore implies that neuronal activity and exosome release are segregated in neuronal cells under physiological conditions, presumably as a consequence of the different effector molecules required for synaptic vesicle versus MVB‐PM fusion and their differential sensitivity to cellular stimuli. Consistent with this view, the application of a HFS induced MVB‐PM fusion events in a small population of neurons (≈10%) (Figure 1d–f). Whereas MVB‐PM fusion followed the HFS with a time lag of several tens of seconds, the same HFS evoked a fast and immediate increase in intracellular calcium (Figure 1g,h). These results thus demonstrate that different from neurotransmitter vesicle fusion, MVB‐PM fusion is not directly coupled to a rise in intracellular calcium and likely involves a complex network events downstream the initiating calcium signal. The results from investigating the effect of store‐derived calcium signals (Figure S2, Supporting Information) further suggests that the calcium signal required for EV release is likely to be more complex than a simple rise in cytosolic calcium. Our results put previous reports, which directly connect MVB‐PM fusion and neuronal exosome release to neuronal activity, in a different perspective.^41^, ^42^, ^43^, ^44^, ^45^ The results from these previous studies were derived from quantifying EVs in the cell culture medium in response to long‐lasting neuronal membrane depolarization without examining the effect of a brief and well‐defined electrical stimulus on MVB‐PM fusion. Thus, these results may be a consequence of using a non‐physiological stimulus. At present, it is unclear, why the effect of HFS is restricted to such a small neuronal population. Akin to presynaptic terminals, where a readily releasable pool (RRP) of transmitter vesicles is required for successful neurotransmitter release,^82^ PM‐docked and therefore releasable MVBs may only be present in a small subset of neurons. In B‐lymphocytes two pools of MVBs can be identified based on their membrane cholesterol content, where only MVBs with high membrane cholesterol levels are able to fuse with the plasma membrane and release exosomes.^83^ Alternatively, stimulus‐responsive MVB‐PM fusion may require the expression of cell‐type specific SNARE‐associated molecules. Because of their particular role for EV biology, we therefore suggest the novel term e‐SNARE for SNARE proteins implicated in the release of exosomes. Our results imply that growth factor stimulate EV release by affecting the molecular machinery required for vesicle fusion, which include SNAREs such as VAMP3 (Figure 5 & 6). The fact that blocking receptor tyrosine kinases by genistein decreases the number of EVs to sub‐normal levels (Figure 4d,e) suggests a baseline activation in cultured neurons by endogenous pleiotropic growth factor support. Because of its unspecific effect on receptor tyrosine kinases, genistein likely affects a number of growth factor signaling networks other than downstream from bFGF receptors, including the classical neurotrophins nerve growth factor (NGF) and brain derived neurotrophic factor (BDNF).^84^ Interestingly, we found these latter molecules to mimic the effect of bFGF on EV release (Figure S8, Supporting Information). These results point toward a broader regulatory role of neurotrophic factors for neuronal EV release and future studies should examine the specific effects of distinct neuronal growth factors on EV release. On the other hand, although growth factors enhance stimulus‐responsive EV release, their role is additive to other factors, since genistein did not entirely block EV release in these cells. In addition, the effect of genistein on EV release exceeded the effect of BAPTA treatment, likely because of its additional off‐target effects.^85^ Future research will need to examine the specific cellular and molecular factors that determine the availability of MVBs for stimulus‐evoked PM fusion and the different populations of releasable MVBs in neuronal cells. In support of such studies, emerging evidence indicates that the coupling of neuronal EV release to neuronal activity could be functionally relevant for plasticity‐associated processes in the CNS.^17^, ^30^, ^31^, ^41^, ^86^, ^87^, ^88^

In the present study, we examined the effect of bFGF on EV release in cultured hippocampal neurons. Our results demonstrate that bFGF stimulates EV release from cultured hippocampal neurons (Figure 4). Our proteomic analysis and molecular biology experiments further suggest bFGF to affect key cellular protein circuits that control vesicle membrane fusion (Figure 5). In summary, our data thus reveal a new and previously unrecognized function of bFGF in controlling neuronal EV release, and implicate the SNARE protein VAMP3 in the bFGF‐mediated effect on EV release. Since MVB‐PM fusion and neurotransmission are both mediated by SNARE proteins and because both are regulated by similar stimuli, the question raises how neurons segregate neurotransmitter release from exosome release. The SNAREs that are used for synaptic vesicle exocytosis are VAMP2 (also called synaptobrevin 2), syntaxin 1a, and SNAP‐25.^89^ In accord, mice lacking VAMP2 have a significantly reduced synaptic transmission (around 10% of control),^81^ emphasizing the role of VAMP2 for synaptic vesicle fusion, where VAMP3 is not required. Our biochemical results (Figure 6) demonstrate a strong signal for VAMP3 in EVs, whereas VAMP2 was not detectable by Western blot, suggesting low abundance. Our results suggest a non‐redundant functional role of VAMP2 and VAMP3 for EV release which may serve as a molecular basis for the segregation of neurotransmitter and stimulus‐evoked EV release in neurons. Although an attractive model, the role of VAMP 3 for EV release is likely to be more complex, because we found that treatment with *Vamp3* siRNA had no consistent effect on the abundance of the EV signature proteins CD81, CD9, Alix or Flotilin‐1 in EV‐enriched cell culture medium pellets (Figure S9, Supporting Information). A number of reasons may account for such inconsistencies between the two different assays. First, bFGF has a profound effect on the abundance of EV‐enriched proteins, as demonstrated by the MS‐based quantification of the ExoCarta top 100 EV‐enriched proteins in bFGF and VEH‐treated neurons (Figure S10 and Table S2, Supporting Information), that is, bFGF affects the abundance of these proteins in each vesicle and independent from its effect on the number of vesicles. Second, recent evidence suggests that various EV subpopulations may exist within a given biological specimen, each of them with a unique protein composition, internal content and function.^90^ Both of these factors fairly contribute to EV complexity in biological fluids and future research thus needs to address the specific effect of bFGF and VAMP3 on distinct EV subpopulations and their protein composition. Moreover, VAMP2 and 3 interact with numerous other syntaxins (Figure 5h), thus adding another layer of complexity.

Our biochemical (Figure S1f, Supporting Information) and NTA data (Figure 4a) suggest that bFGF predominantly affects the abundance of exosomes over other types of EVs. To confirm this hypothesis, we applied pH‐sensitive dye imaging where pHluorin is tagged to the EV‐enriched tetraspanin CD63. In line with our biochemical data, we found that treatment with bFGF increased the stimulus‐evoked increase in pHluorin fluorescence (Figure 2), thus further demonstrating that bFGF stimulates the release of exosomes over other types of EVs. The latter interpretation is however mitigated by restriction of the imaging method we applied: since we used patch‐clamp electrophysiology in conjunction with conventional epifluorescence microscopy for imaging CD63‐pHluorin instead of using total internal reflection microscopy (TIRF), an increase in fluorescence could be a result of MVB‐PM fusion and exosome release, or alternatively, be due to an abrupt, stimulus‐induced increase in the pH‐value inside CD63‐tagged MVB in the absence of MVB‐PM fusion. We are aware of these concerns but favor the former explanation because i) bFGF treatment clearly results in an increased abundance of EVs in the cell culture medium (Figure 4a,b) and ii) because treatment with bFGF rather decreases the pH‐value inside CD63‐tagged MVBs under unstimulated conditions (Figure 3a–e). In fact, it would be even tempting to speculate about an increased EV release as a result of a decreased endo‐lysosomal pH‐value, similar to what has been shown for synaptic vesicles,^74^ although this hypothesis clearly deserves further investigation. Although our data favor a loose coupling between calcium and EV release, we found bFGF‐stimulated exosome release to be calcium‐dependent after prolonged (24 h) treatment, as BAPTA‐AM normalized the effect of bFGF (Figure 4g–i) and on the other hand bFGF increased cytoplasmatic calcium concentrations (Figure 3f), presumably as a result of an activation of voltage‐independent calcium stores.^77^, ^78^, ^79^ Moreover, epifluorescence imaging allowed us to capture a wider area including the entire soma and dendritic arbor, where TIRF is restricted to a small area due to the curved geometry of neuronal cells.

Although calcium has become a common theme for exosome release, other studies found MVB‐PM fusion to be independent from intracellular calcium in HeLa cells.^20^ Future studies will need to examine the specific role of the intracellular neuronal calcium domains for the effect of bFGF on MVB‐PM fusion. In principal, increase in exosomes is due to an enhanced MVB‐PM fusion, or be the consequence of an increased MVB size or number. Our data point toward an effect of bFGF on MVB availability or fusion rate because we found no difference in the size or number of MVBs, although there was a trend towards more and bigger MVBs in our EM analysis (Figure S5, Supporting Information). This is in contrast to previous reports, demonstrating the volume of MVBs to increase in response to treatment with the neurotrophin prosaposin^91^ or when the neurotrophin receptor TrkB is overexpressed.^92^

Although providing new insights into the molecular mechanism of neuronal exosome release, the translational potential of our results is limited by the lack of in vivo data. Therefore, future studies will have to confirm our results in vivo, for example, by using suitable reporter animal models that have been published recently.^25^ It would be interesting to determine, if EV release exhibits short‐ or long‐term changes through electrical stimulation in vivo, like this is known to be the case for hippocampal synaptic transmission (i.e., long‐term potentiation). Such in vivo or ex vivo experiments will provide further insight into EV‐mediated cell‐to‐cell communication in the intact CNS, although adding another layer of complexity that comes with the presence of different cell types in the CNS and thus making these experiments technically more challenging.

## 4 Conclusion

In summary, our results thus reveal a new function for neurotrophic factor signaling in controlling activity‐dependent neuronal EV release and support the investigation of growth factor‐mediated signal transduction via EVs. Our results aid the examination of growth factor‐mediated EV release in pathological conditions, where EVs contribute to the progression of neurodegenerative diseases, such as Parkinson's disease (PD) or Alzheimer's disease (AD).^93^, ^94^, ^95^, ^96^, ^97^ Furthermore, our results will promote the investigation of EV‐associated changes in responses to neuronal injury^48^, ^49^, ^50^ or psychiatric conditions where bFGF has been shown to be strongly implicated (reviewed in refs. 51, 52).

## 5 Experimental Section

##### Cell Culture

Animals were handled in accordance with the regulations of local authorities and the animal welfare committee of the Ludwig Maximilian University Munich, Germany. Rat hippocampal neurons were prepared from embryonic day 18 CD rats (Charles River) and cultivated in Neurobasal media containing 2% B27, 0.25% glutamine, and 0.125% glutamate (Invitrogen) as described before.^98^ Unless otherwise indicated, neurons were treated with 50 ng mL^−1^ basic fibroblast growth factor (Peprotech) diluted in PBS.

##### Lentiviral Production and Virus Transduction

CD63‐pHluorin, where pHluorin is cloned into the second luminal loop of mouse CD63, was expressed from a modified FUGW lentiviral vector under the control of the neuron‐specific human synapsin promoter (*p*CD63‐pHluorin). The generation of the plasmid is described in detail elsewhere.^74^ *p*CD63‐pHluorin was obtained from Dr. Benjamin Rost (German Centre for Neurodegenerative Research, Berlin, Germany). Viral particles were produced as described before.^99^ Unless described otherwise, rat hippocampal neurons were transduced with CD63‐pHluorin lentiviral particles at day 3 in vitro (DIV). After 24 h, the medium was removed and the cells washed three times with PBS and fresh medium was replenished.

##### Small Interfering RNA Transfection

Small interfering RNAs (siRNAs) (Silencer Select siRNAs, Thermo Fisher Scientific) targeting VAMP3 (#4 390 771) or control siRNAs (#4 390 843) were mixed with Lipofectamine RNAi Max according to the manufacturer's recommendations. The siRNAs were applied at DIV7 at a final concentration of 10 nm and the cells were incubated with the siRNAs for the following 2 days until DIV 9, the cells were harvested for Western blot and the medium collected for NTA.

##### Live Cell Microscopy

All live cell imaging experiments were performed on an inverted Leica DMI6000 B microscope controlled via Leica Application Suite X (LAS X) software. For CD63‐pHluorin imaging, hippocampal neurons, cultured on glass bottom cell culture dishes (Ibidi), were transferred to the microscope stage at DIV 12–14 and perfused with ACSF containing the following: 140 mm NaCl, 2.4 mm KCl, 10 mm Hepes, 10 mm glucose, 4 mm CaCl_2_, and 4 mm MgCl_2_, 320 mOsmol/L, pH 7.4. To visualize pHluorin fluorescence, the cells excited with a UV light source equipped with a 488 nm excitation filter and imaged at 1 frame per second by a CD camera (Hamamatsu). Ammonium chloride (NH_4_Cl) was applied at a concentration of 50 mm diluted in ACSF through a ValveLink8.2 perfusion system (AutoMate). Fura‐2 AM calcium imaging was performed as described before.^100^ Leica Application Suite X (LAS X) software Graph Pad Prism 7 were used for data analysis.

##### Preparation of EV‐Enriched Pellets from Neuronal Cell Culture Medium

To prepare EV‐enriched pellets, cell culture medium was changed at DIV 11 and collected after 24 h (5 mL medium/500 000 cells). EVs were isolated by differential ultracentrifugation as previously described^5^ with minor modifications. In brief, the conditioned medium was centrifuged at 300 × *g* for 10 min at 4 °C to pellet cells. The supernatant was centrifuged at 1 000 × *g* for 20 min at 4 °C, transferred to new tubes, and centrifuged in a MLA‐80 rotor (Beckman) for 90 min at 100 000 × *g* to obtain a 100 K pellet. All pellets were washed in 1 mL of PBS and re‐centrifuged at the same speed in a TLA‐55 rotor (Beckman) before being re‐suspended in 35 µL of sterile PBS or otherwise in 35 µL RIPA buffer. The resulting pellet was defined as EV‐enriched medium pellet.

##### Iodixanol Gradient Separation

Iodixanol gradients were prepared as described.^5^ In brief, EV‐enriched medium pellets (20 mL medium/1‐2 mio. cells) were washed and resuspended in 1.5 mL buffer containing: 0.25 m sucrose, 10 mm Tris pH 8.0, 1 mm EDTA (pH 7.4), and mixed 1:1 with 60% (w/v) stock solution of iodixanol/Optiprep. A 40% iodixanol working solution was prepared and used to prepare 20% and 10% (w/v) iodixanol solutions. Next, 1.3 mL 20% (w/v) iodixanol and 1.2 mL 10% iodixanol were layered on top of the EV suspension and tubes were centrifuged for 1 h at 4 °C at 350 000 × *g* in a SW55Ti rotor (Beckman). Ten fractions of 490 µL were collected from the top of the tube. The density was assessed with a refractometer and the fractions were diluted with 2.5 mL PBS and centrifuged for 30 min at 100 000 × *g* in a TLA 110 rotor (Beckman). These concentrated fractions were resuspended in 30 µL of RIPA and separated on gels.

##### Western Blot

Cultured neurons or EV‐enriched pellets were lyzed in RIPA buffer incl. a protease and phosphatase inhibitor cocktail (Complete Protease Inhibitor Cocktail, PhosStop Phosphatase Inhibitor Cocktail, both Roche). The lysates were incubated for 30 min on ice and centrifuged at 5000 × *g* for 15 min at 4 °C, and the supernatant retrieved. Protein concentrations were determined using the BCA method (Thermo Fisher Scientific) and a spectrophotometer (NanoDrop, Thermo Fisher Scientific). After adjusting protein concentrations, protein lysates were heated to 75 °C for 15 min in Laemmli sample buffer containing 10% beta‐mercaptoethanol. SDS‐PAGE was performed by using Mini‐Protean TGX Gels (Bio‐Rad) and a Tris‐glycine‐based running buffer. The protein was blotted onto polyvinylidene difluoride (PVDF) membranes (Bio‐Rad) at 150 mA for 60–120 min on ice. The membranes were blocked with 5% dry milk in TBST wash buffer (tris‐buffered saline containing 0.05% tween) for 1 h and incubated at 4 °C overnight under gentle shaking with the primary antibody in TBST/5% BSA (Cell Signaling Technology). The membranes were washed and incubated with the respective HRP‐conjugated secondary antibody (Vector Labs) in TBST/5% milk for 1 h, followed by further washing and exposure to Clarity Western blot ECL Substrate (Bio‐Rad) or ECL Prime (GE Healthcare). Chemiluminescence was detected with LI‐COR Odyssey Fc Imaging system and analyzed by Image Studio software (Licor). Western blot images were further processed by ImageJ and the optical densities of target proteins were scaled to the respective loading controls for statistical analysis. Therefore, a normalized experimental signal (measured experimental signal/lane normalization factor) was used, where the lane normalization factor was calculated from the ratio between the measured signal of a housekeeping protein for each lane divided by the highest measured signal of housekeeping protein on the blot. For quantifying exosomal proteins by Western blot, the signal was normalized to the protein concentration of the cell lysate.

##### Reagents and Antibodies

The following primary antibodies were used for Western blot and ICC: anti‐GFP (GTX113617, GeneTex), anti‐Alix/AIP1 (ABC40, Merck), anti‐CD81 (sc‐166029, Santa Cruz Biotechnology), rabbit anti‐rat Rab5 (C8R1, Cell Signaling Technology), rabbit anti‐rat Rab7 (D95F2, Cell Signaling Technology), anti‐Rab11 (D4F5, Cell Signaling Technology), rabbit anti‐rat EEA1 (C45B10, Cell Signaling Technology), anti‐LBPA (Z‐PLBPA, Echelon Biosciences), anti‐CHC (D3C6, Cell Signaling Technology). The following chemical compounds and growth factors were used: BAPTA‐AM (Tocris), Genistein (Tocris), EGTA (Tocris), bFGF (Peprotech), Poly‐l‐Lysine (Sigma), Tetrodotoxin (TTX) (Abcam), Tetraethylammonium chloride (TEA) (Abcam).

##### Immunocytochemistry and Confocal Microscopy

For ICC, cultured neurons were fixed for 15 min at room temperature in 4% paraformaldehyde, washed once for 10 min in PBS, and permealized for 1 min with 0.125% Triton X‐100 in PBS. Unspecific antibody binding was blocked by 1% bovine serum albumin in PBS for 1 h followed by incubation with the respective primary antibody at 4 °C overnight. Unbound antibodies were removed by washing with PBS and the cells were incubated with Alexa Fluor‐conjugated secondary antibodies for 1 h at room temperature. Unbound secondary antibodies were removed by washing in PBS and the cells were air‐dried, covered with fluorescence mounting medium (Dako), and sealed under cover glass. Images were captured using a Leica SP5 confocal microscope and analyzed using Fiji software (https://fiji.sc/Fiji).

##### Electrophysiology and Imaging

All electrophysiological experiments were performed on rat hippocampal neurons at DIV 12–14 grown on glass coverslips (German Glass) pre‐treated with 65% nitric acid and coated with 1 mg mL^−1^ Poly‐d‐Lysin (Sigma). Experiments were performed on an upright Olympus BX51 microscope equipped with a Heka EPC 10 USB amplifier (Heka) under the control of Patchmaster 10 software (Heka). Intracellular (pipette) solution contained the following: 136 mm KCl, 17.8 mm Hepes, 1 mm EGTA, 0.6 mm MgCl_2_, 4 mm NaATP, 0.3 mm Na_2_GTP, 15 mm creatine phosphate, and 5 U mL^−1^ phosphocreatine kinase, 315–320 mOsmol L^−1^, pH 7.4. Extracellular solution contained the following: 140 mm NaCl, 2.4 mm KCl, 10 mm Hepes, 10 mm glucose, 4 mm CaCl_2_, and 4 mm MgCl_2_, 320 mOsmol L^−1^, pH 7.4; patch‐pipette solution 136 mm KCl, 17.8 mm Hepes, 1 mm EGTA, 0.6 mm MgCl_2_, 4 mm NaATP, 0.3 mm Na_2_GTP, 15 mm creatine phosphate, and 5 U mL^−1^ phosphocreatine kinase, 315–320 mOsmol L^−1^, pH 7.4. Electrophysiological data were analyzed with Axograph X version 1.6.5. (Axon Instruments). Live‐cell imaging videos in conjunction with patch‐clamp experiments were captured time locked to an electric stimulus by using a mounted CD camera (ExiBlue, QImaging) controlled by µManager software at a frame rate of 10 frame per second.

##### Nano‐Particle Tracking Analysis

For NTA experiments, the cell culture medium was changed at DIV 11 and collected after 24 h (1.5 mL medium/150 000 cells). A NanoSight LM10 NTA apparatus (Malvern) was used for all NTA measurements. For each sample, at least three videos of 60 s with more than 200 detected tracks per video, and in at least one dilution, were taken and analyzed using the NanoSight LM10 NTA software v3.00 and Graph Pad Prism 7.

##### Single Cell Imaging

For local (single cell) calcium measurements, neurons were filled with 0.05 mm Oregon Green 488 BAPTA‐1 AM (Life Technology) in the patch‐pipette solution over a period of 2–2.5 min. Captured videos were edited and analyzed using Fiji (ImageJ) and Graph Pad Prism 7.

##### Quantification of Neuronal Cell Death

Cell death in cultured neurons was quantified as described before.^101^ In brief, lactate dehydrogenase (LDH) released into the culture medium was measured using the CytotoxOne Membrane Integrity Assay (Promega) according to the manufacturer's instructions with a Fluostar Omega fluorescence reader (BMG Labtech).

##### Electron Microscopy

For EM, rat hippocampal neurons at DIV 12–14 were grown on glass coverslips (German Glass), pre‐treated with 65% nitric acid, and coated with 1 mg mL Poly‐d‐Lysin (Sigma). The medium was removed, the cells washed three times with PBS, and pre‐fixed for 5 min with 4% PFA. After this, they were washed three times with 75 mm cacodylate buffer including 150 mm NaCl and 2 mm MgCl_2_ (fixation buffer) and post‐fixed with 0.2% osmium tetroxide. The cells were again washed once with buffer and three times with H_2_O_bidest_. Dehydration was carried out in a graded acetone series. The 20% acetone step additionally included 1% uranyl acetate. Finally, the cells were embedded in the epoxy resin Epon 812. In general, embedding of the cells, sectioning, and post‐staining were carried out as described.^102^ Transmission electron microscopy was performed on a Zeiss EM 912 equipped with an integrated OMEGA energy filter and operated at 80 kV in the zero‐loss mode and the images analyzed by Fiji (ImageJ) software.

##### LC‐MS/MS Analysis

The EV‐enriched pellets were lyzed in 80 µL of a modified RIPA lysis buffer (50 mm TrisHCl pH 8, 150 mm NaCl, 5 mm EDTA, 1% v/v Triton X‐100, 0.5% w/v sodium deoxycholate, 0.1% w/v) with protease inhibitors (Sigma‐Aldrich, USA) on ice with intermediate vortexing. 20 µL H_2_O, 10 µL 100 mm MgCl_2_, and 25 units Benzonase (Sigma‐Aldrich, USA) were added followed by an incubation for 30 min at 37 °C at 1400 rpm in a Thermomixer (Eppendorf). Undissolved material was removed by centrifugation for 5 min at 20 000 *g* and 4 °C. The supernatants were transferred to 1.5 mL protein Lobind Tubes. Proteins were reduced by addition of 9 µL of 200 mm dithiothreitol (Biozol) in 50 mm ammonium bicarbonate and incubation for 30 min at 37 °C. Cysteine residues were alkylated by addition of 18 µL 400 mm iodoacetamide (Sigma) and incubation for 30 min at room temperature in the dark. Afterward, the reaction was quenched by adding another 9 µL of 200 mm dithiothreitol. Proteolytic digestion was performed using a modified protocol for single‐pot solid‐phase enhanced sample preparation (SP3).^103^ Briefly, after binding of proteins to 40 µg of a 1:1 mixture of hydrophilic and hydrophobic magnetic Sera‐Mag SpeedBeads (GE Healthcare) with a final concentration of 70% acetonitrile for 30 min at room temperature, beads were washed twice with 200 µL 70% ethanol and twice with 180 µL acetonitrile. For proteolytic digestion, 125 ng LysC and 125 ng trypsin (Pomega) were added in 20 µL 50 mm ammonium bicarbonate followed by an incubation for 16 h at room temperature. The supernatants were transferred to fresh 0.5 mL protein lobind tubes (Eppendorf). For improved peptide recovery, 20 µL 0.1% formic acid were added to the magnetic beads followed by sonication for 30 s in a sonication bath (Hielscher Ultrasonics GmbH). The supernatants were combined and dried by vacuum centrifugation. Peptides were re‐dissolved in 20 µL 0.1% formic acid. The peptide concentration was estimated using Nanodrop at 280 nm (Thermo).

The peptides were analyzed on an Easy nLC 1000 nanoHPLC (Thermo Scientific) which was coupled online via a Nanospray Flex Ion Source (Thermo Sientific, USA) equipped with a PRSO‐V1 column oven (Sonation) to a Q‐Exactive mass spectrometer (Thermo Scientific). An amount of 1 µg of peptides per sample was separated on an in‐house packed C18 column (30 cm × 75 µm ID, ReproSil‐Pur 120 C18‐AQ, 1.9 µm, Dr. Maisch GmbH) using a binary gradient of water i) and acetonitrile ii) supplemented with 0.1% formic acid (0 min, 2% B; 3:30 min, 5% B; 137:30 min, 25% B; 168:30 min, 35% B; 182:30 min, 60% B) at 50 °C column temperature. A data‐dependent acquisition method was used. Full MS scans were acquired at a resolution of 70 000 (*m*/*z* range: 300–1400, AGC target: 3E+6). The ten most intense peptide ions per full MS scan were selected for peptide fragmentation (resolution: 17 000, isolation width: 2 *m*/*z*, AGC target: 1E+5, NCE: 25%). A dynamic exclusion of 120 s was used for peptide fragmentation. The raw data were analyzed with the software Maxquant (maxquant.org, Max‐Planck Institute Munich) version 1.6.1.0.^104^

The MS data were searched against a canonical fasta database of Rattus norvegicus from UniProt (download: March 5, 2018, 29 975 entries). Trypsin was defined as protease. Two missed cleavages were allowed for the database search. The option first search was used to recalibrate the peptide masses within a window of 20 ppm. For the main search peptide and peptide fragment mass tolerances were set to 4.5 and 20 ppm, respectively. Carbamidomethylation of cysteine was defined as static modification. Acetylation of the protein N‐term as well as oxidation of methionine was set as variable modifications. The false discovery rate for both peptides and proteins was adjusted to less than 1%. Label free quantification (LFQ) of proteins required at least two ratio counts of razor peptides. Only razor and unique peptides were used for quantification. The protein LFQ intensities were log_2_ transformed and a two‐sided Student's *t*‐test was applied to evaluate the significance of proteins with changed abundance. Additionally, a permutation based false discovery rate estimation was used.^105^

##### Network Architecture and Centrality Analysis

Raw MS data were analyzed using R statistical environment version i386 3.3.3 (http://www.r‐project.org./). A hierarchical cluster analysis was performed using Euclidean similarity metric in R and clustered heat‐maps were visualized using the Heatmap3 R package. In order to check for the reproducibility of the array replicates Pearson's correlation analysis was performed using R and plotted as heat‐maps. In order to overview the array replicates distribution and segregation PCA was performed using “procomp” function of the stat package in the R statistical environment.

Accession IDs of 2258 protein hits from (MS) data were used to obtain protein information and gene IDs using Uniprot database^106^ and were selected for further pathway analysis. Both up‐regulated and down‐regulated peptide hits with ±1.5‐fold enrichment in MS data were further considered for pathway enrichment analysis. METASCAPE^107^ (https://metascape.org) was used in order to perform pathway enrichment analysis; which contains Kyoto Encyclopedia of genes and genomes (KEGG). Pathways were considered statistically significant using a *p*‐value ≤ 0.05. Consideration for further analysis was given to the candidates contributing to enrichment of the pathways reported in vesicle mediated transport, regulation of vesicle mediated transport, and import to the cell.

Protein–protein interactions (PPINs) were retrieved by using STRING database.^108^ A parameter of high confidence (0.7) was set for functional interaction among the candidates of interest and an intereactome among candidate proteins was obtained. The intereactome maps obtained from STRING database were visualized by using Cytoscape v3.6.1 (https://www.cytoscape.org/)^109^ and hub proteins were obtained through centrality analysis.

## Conflict of Interest

The authors declare no conflict of interest.

## Supporting information

Supporting Information

Supplemental Table S1

Supplemental Table S2

Supplemental Movie 1

Supplemental Movie 2a

Supplemental Movie 2b

Supplemental Movie 3a

Supplemental Movie 3b

Supplemental Movie 3c

Supplemental Movie 3d
