# Supplementary material for: Fibroblast Growth Factor 2‐Mediated Regulation of Neuronal Exosome Release Depends on VAMP3/Cellubrevin in Hippocampal Neurons
Source: Adv Sci (Weinh). 2020 Jan 28;7(6):1902372. doi: 10.1002/advs.201902372 (PMC7080514; doi:10.1002/advs.201902372)
Supplement: Supplementary file 1 — Supporting Information [file ADVS-7-1902372-s001.pdf]

Copyright WILEY-VCH Verlag GmbH & Co. KGaA, 69469 Weinheim, Germany, 2020.

ADVANCED SCIENCE
Open Access

Supporting Information

for *Adv. Sci.,* DOI: 10.1002/advs.201902372

Fibroblast Growth Factor 2-Mediated Regulation of Neuronal Exosome Release Depends on VAMP3/Cellubrevin in Hippocampal Neurons

*Rohit Kumar, Qilin Tang, Stephan A. Müller, Pan Gao, Diana Mahlstedt, Sofia Zampagni, Yi Tan, Andreas Klingl, Kai Bötzel, Stefan F. Lichtenthaler, Günter U. Höglinger, and Thomas Koeglsperger**

Supporting Information:

Fibroblast Growth Factor 2-mediated Regulation of Neuronal Exosome Release Depends on VAMP3/cellubrevin in Hippocampal Neurons.

*Rohit Kumar, Qilin Tang, Stephan A. Müller, Pan Gao, Diana Mahlstedt, Sofia Zampagni, Yi Tan, Andreas Klingl, Kai Bötzel, Stefan F. Lichtenthaler, Günter Höglinger & Thomas Koeglsperger**

Figure S1

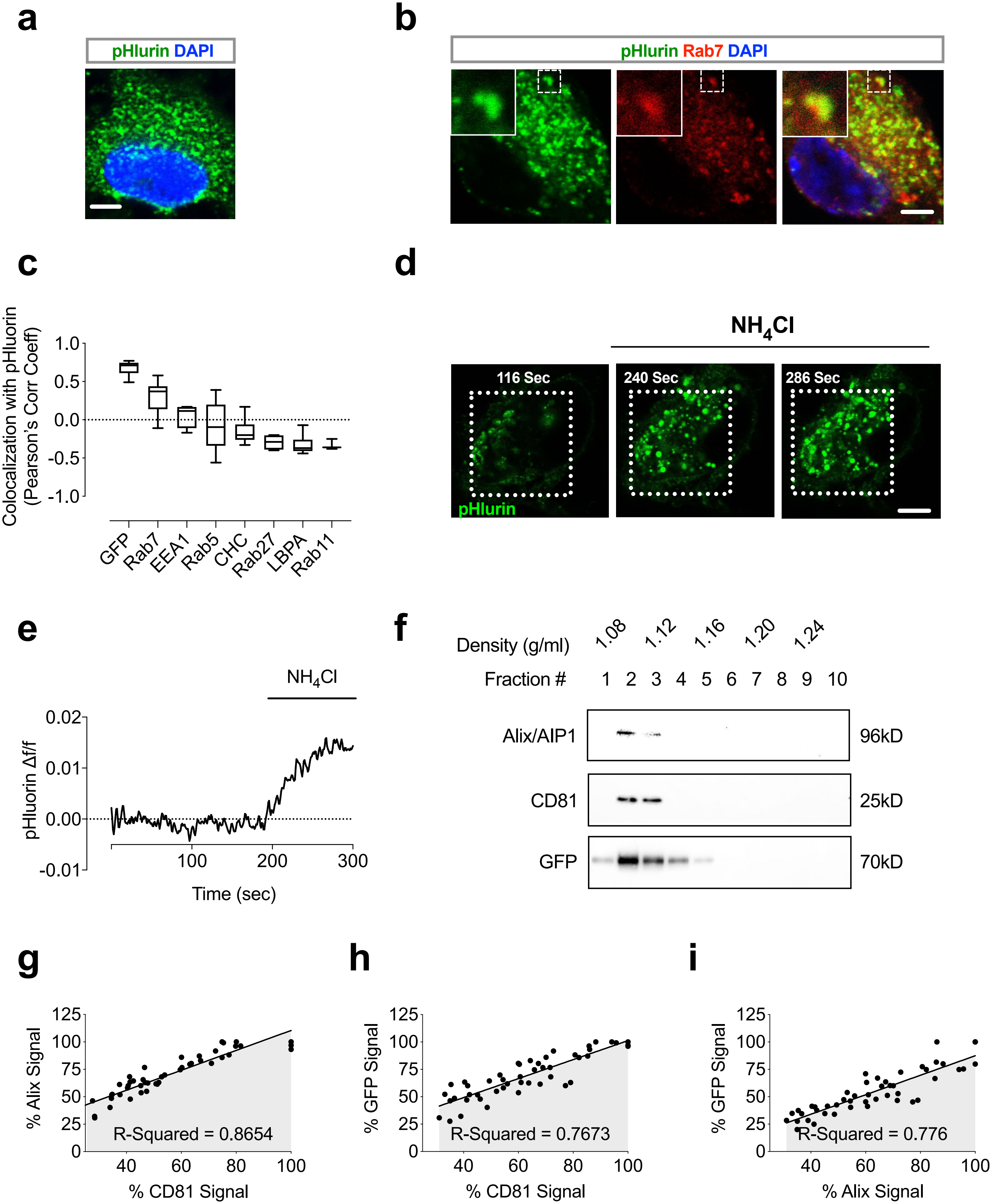
a
pHlurin DAPI
b
pHlurin Rab7 DAPI
c
Colocalization with pHluorin (Pearson's Corr Coeff)
1.0
0.5
0.0
-0.5
-1.0
GFP
Rab7
EEA1
Rab5
CHC
Rab27
LBPA
Rab11
d
NH4Cl
116 Sec
240 Sec
286 Sec
pHlurin
e
NH4Cl
pHluorin Δf/f
0.02
0.01
0.00
-0.01
100
200
300
Time (sec)
f
Density (g/ml)
1.08
1.12
1.16
1.20
1.24
Fraction #
1 2 3 4 5 6 7 8 9 10
Alix/AIP1
96kD
CD81
25kD
GFP
70kD
g
% Alix Signal
% CD81 Signal
R-Squared = 0.8654
h
% GFP Signal
% CD81 Signal
R-Squared = 0.7673
i
% GFP Signal
% Alix Signal
R-Squared = 0.776
125
100
75
50
25
0
40
60
80
100

Figure S2

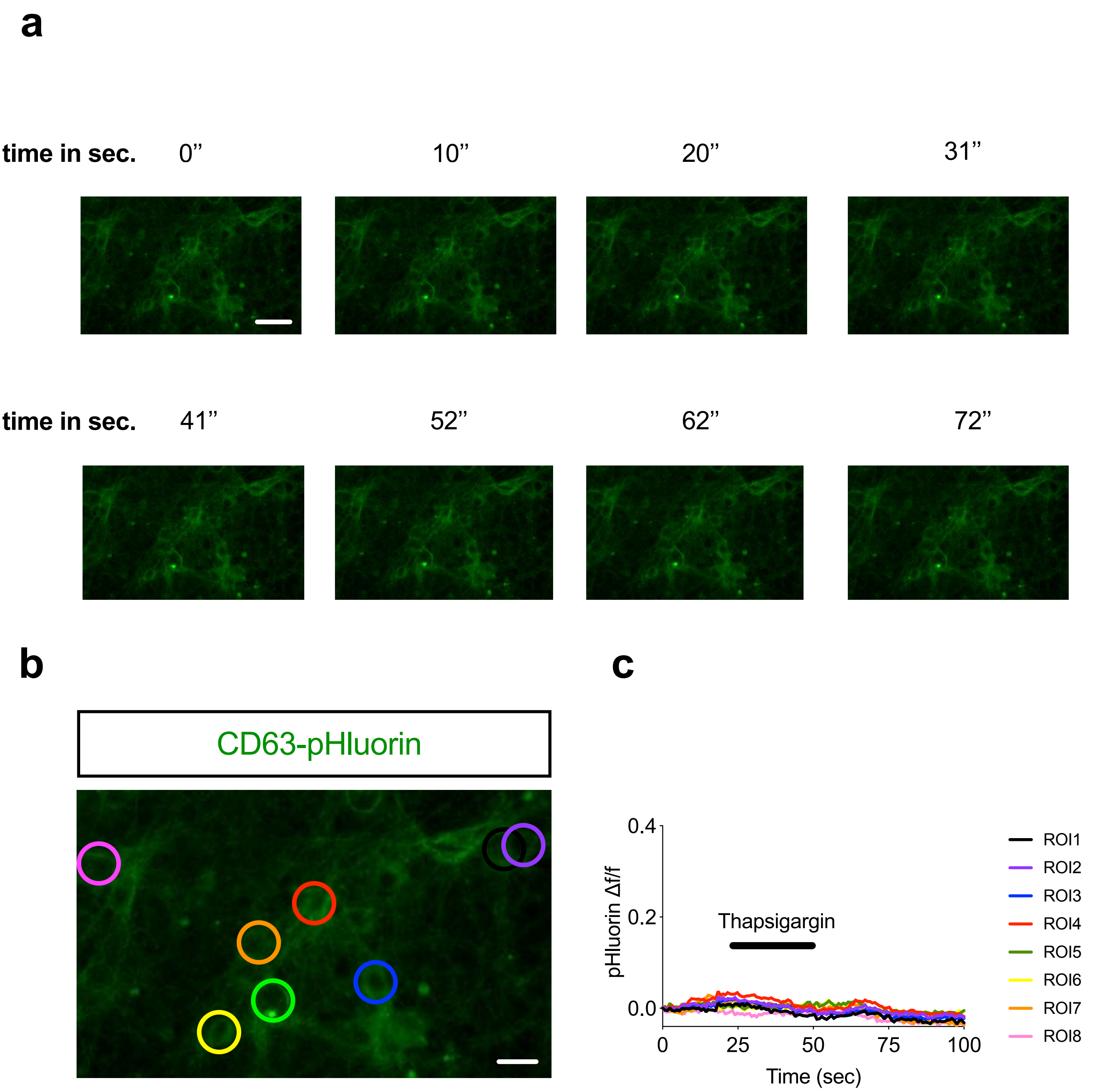
a
time in sec.
0''
10''
20''
31''
time in sec.
41''
52''
62''
72''
b
CD63-pHluorin
c
pHluorin Δf/f
0.4
0.2
0.0
Thapsigargin
0
25
50
75
100
Time (sec)
ROI1
ROI2
ROI3
ROI4
ROI5
ROI6
ROI7
ROI8

Figure S3

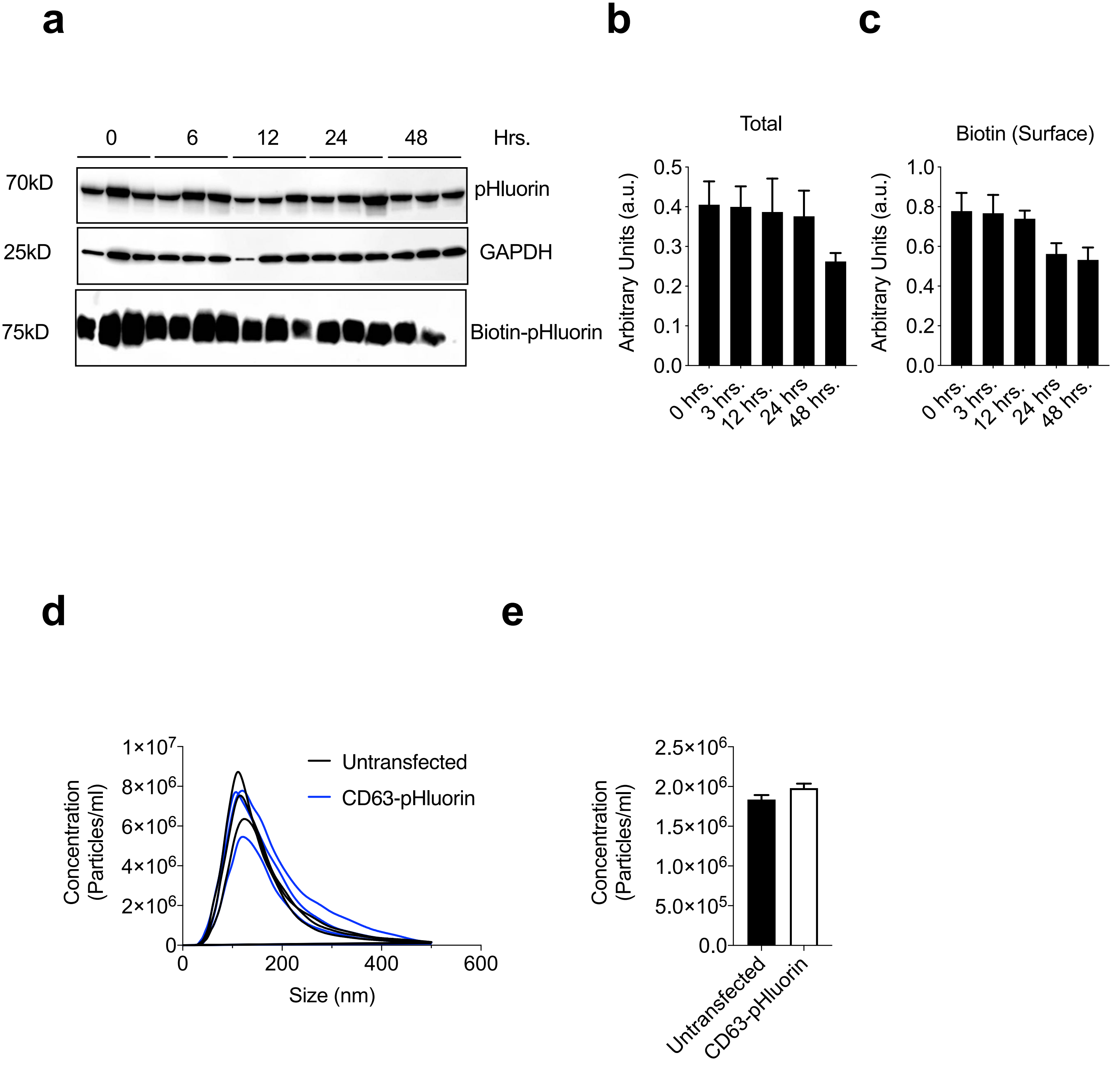
a
0
6
12
24
48
Hrs.
70kD
pHluorin
25kD
GAPDH
75kD
Biotin-pHluorin
b
Total
Arbitrary Units (a.u.)
0.0
0.1
0.2
0.3
0.4
0.5
0 hrs.
3 hrs.
12 hrs.
24 hrs
48 hrs.
c
Biotin (Surface)
Arbitrary Units (a.u.)
0.0
0.2
0.4
0.6
0.8
1.0
0 hrs.
3 hrs.
12 hrs.
24 hrs
48 hrs.
d
Concentration (Particles/ml)
0
2×10^6
4×10^6
6×10^6
8×10^6
1×10^7
Untransfected
CD63-pHluorin
0
200
400
600
Size (nm)
e
Concentration (Particles/ml)
0.0
5.0×10^5
1.0×10^6
1.5×10^6
2.0×10^6
2.5×10^6
Untransfected
CD63-pHluorin

Figure S4

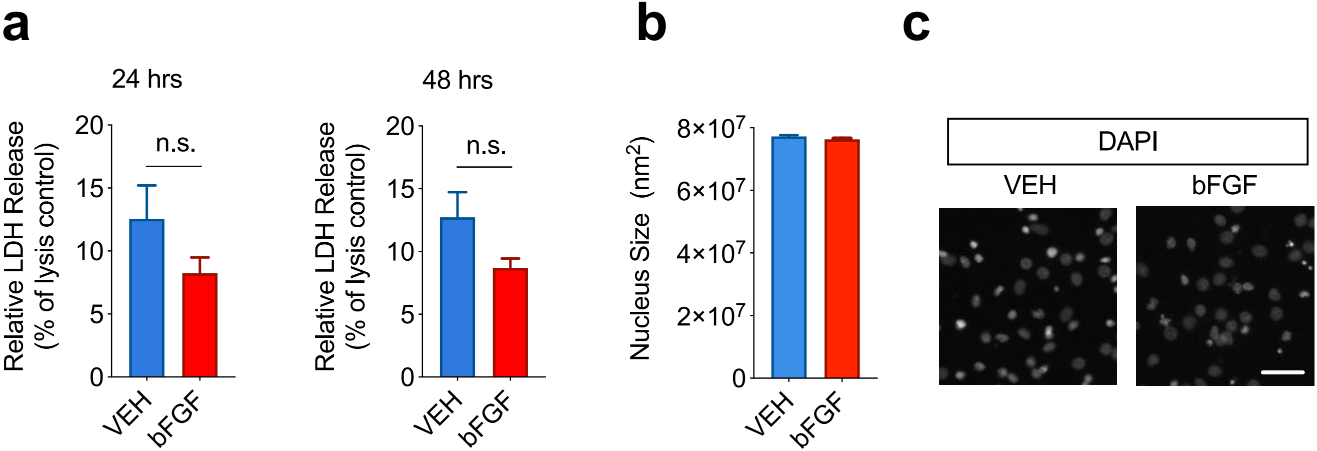

a
24 hrs
48 hrs
Relative LDH Release (% of lysis control)
n.s.
20
15
10
5
0
VEH
bFGF
b
Nucleus Size (nm2)
8×107
6×107
4×107
2×107
c
DAPI
VEH
bFGF

Figure S5

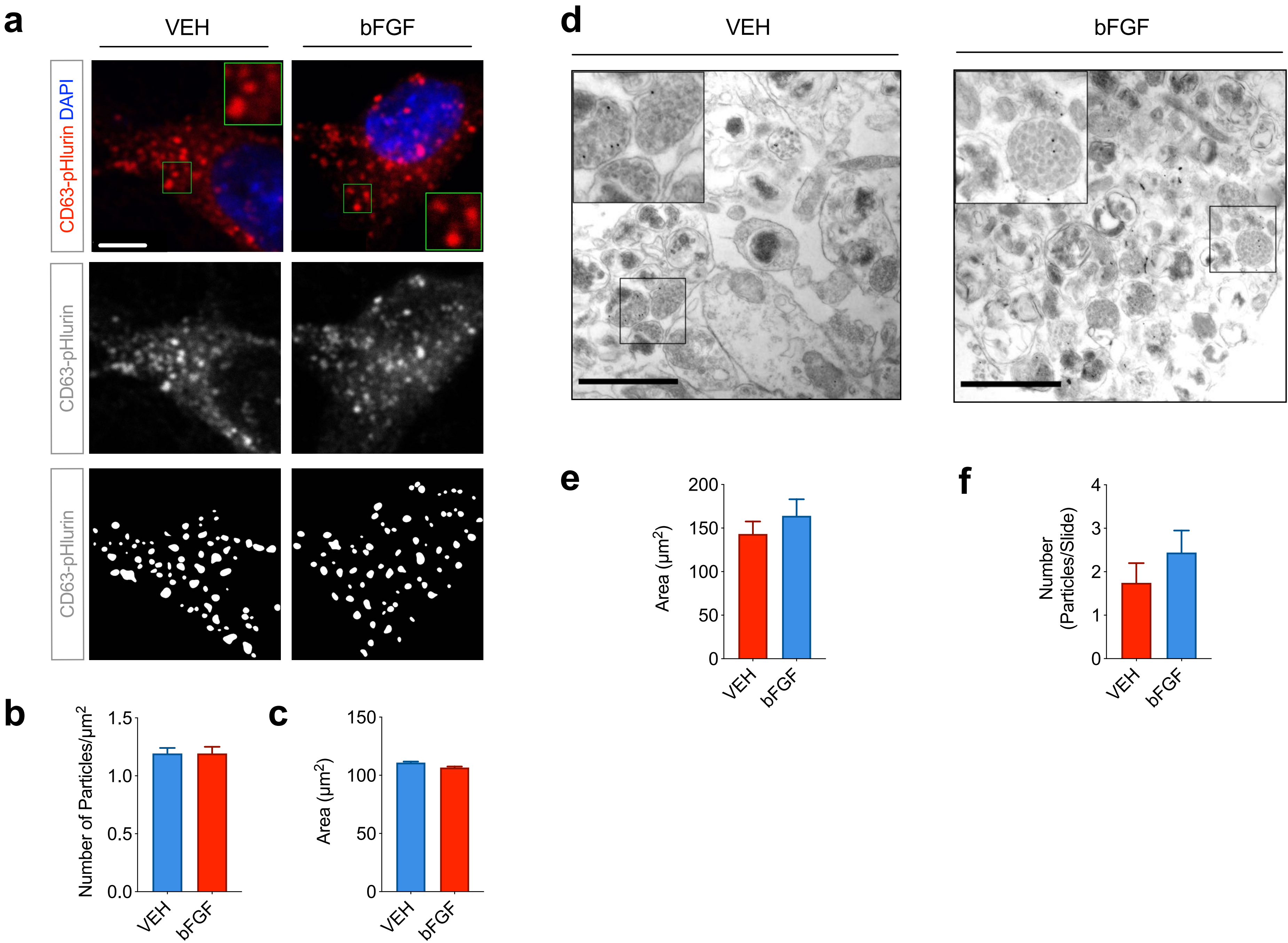
a
VEH
bFGF
CD63-pHlurin DAPI
CD63-pHlurin
CD63-pHlurin
b
Number of Particles/µm2
1.5
1.0
0.5
0.0
VEH
bFGF
c
Area (µm2)
150
100
50
0
VEH
bFGF
d
VEH
bFGF
e
Area (µm2)
200
150
100
50
0
VEH
bFGF
f
Number (Particles/Slide)
4
3
2
1
0
VEH
bFGF

Figure S6

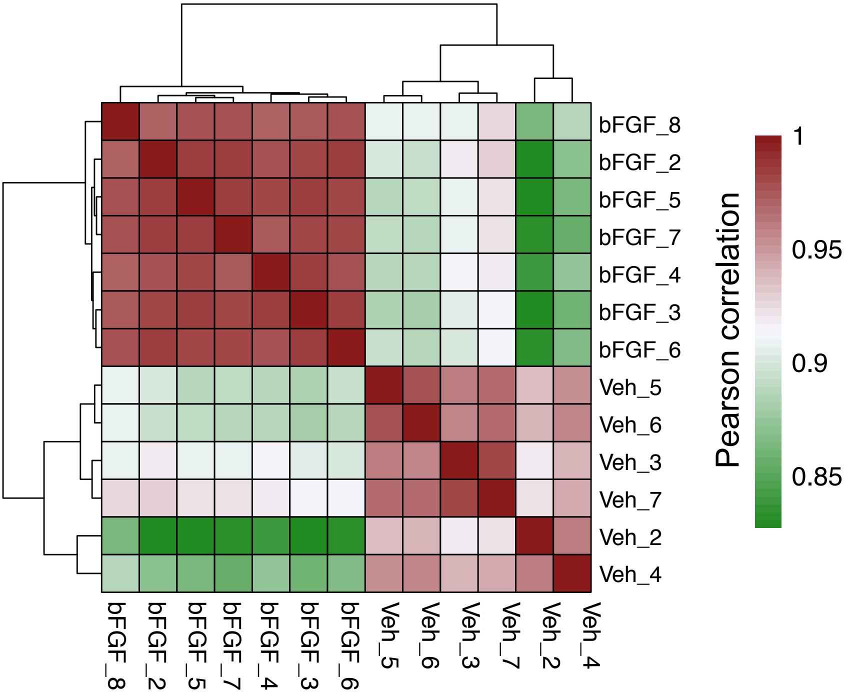

bFGF_8
bFGF_2
bFGF_5
bFGF_7
bFGF_4
bFGF_3
bFGF_6
Veh_5
Veh_6
Veh_3
Veh_7
Veh_2
Veh_4
Pearson correlation
1
0.95
0.9
0.85

Figure S7

a

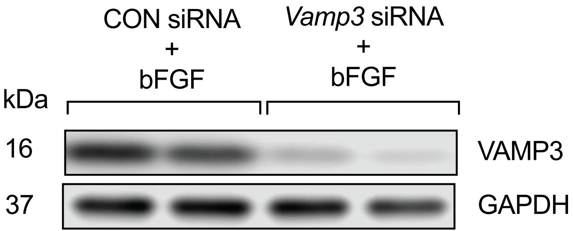
CON siRNA
+
bFGF
Vamp3 siRNA
+
bFGF
kDa
16
37
VAMP3
GAPDH

b

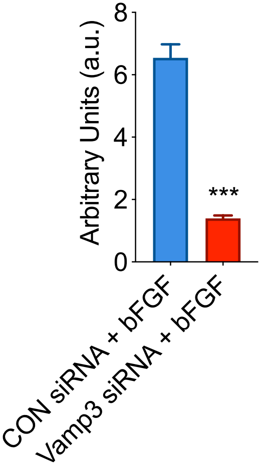
Arbitrary Units (a.u.)
8
6
4
2
0
***
CON siRNA + bFGF
Vamp3 siRNA + bFGF

Figure S8

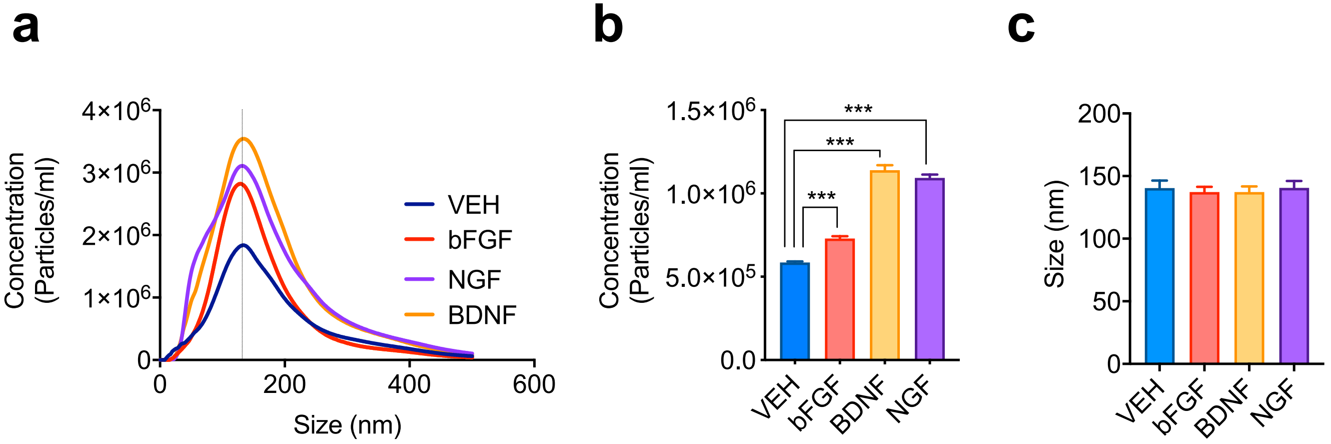
a
b
c
Concentration (Particles/ml)
4×10^6
3×10^6
2×10^6
1×10^6
0
0
200
400
600
Size (nm)
VEH
bFGF
NGF
BDNF
1.5×10^6
1.0×10^6
5.0×10^5
0.0
***
***
***
VEH
bFGF
BDNF
NGF
Size (nm)
200
150
100
50
0
VEH
bFGF
BDNF
NGF

Figure S9

a

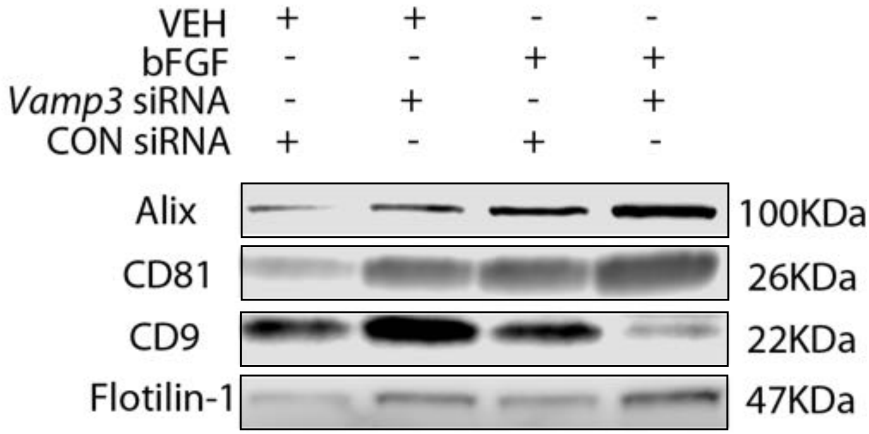

VEH + + - -
bFGF - - + +
Vamp3 siRNA - + - +
CON siRNA + - + -
Alix
CD81
CD9
Flotilin-1
100KDa
26KDa
22KDa
47KDa

b

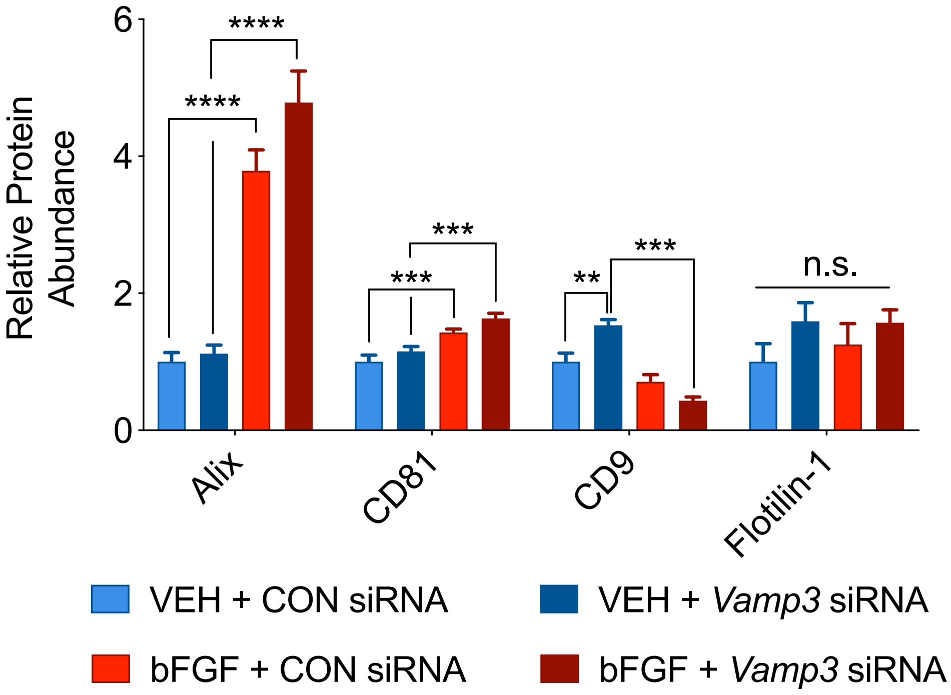

Relative Protein Abundance
0
2
4
6
****
****
***
***
**
***
n.s.
Alix
CD81
CD9
Flotilin-1
VEH + CON siRNA
VEH + Vamp3 siRNA
bFGF + CON siRNA
bFGF + Vamp3 siRNA

Figure S10

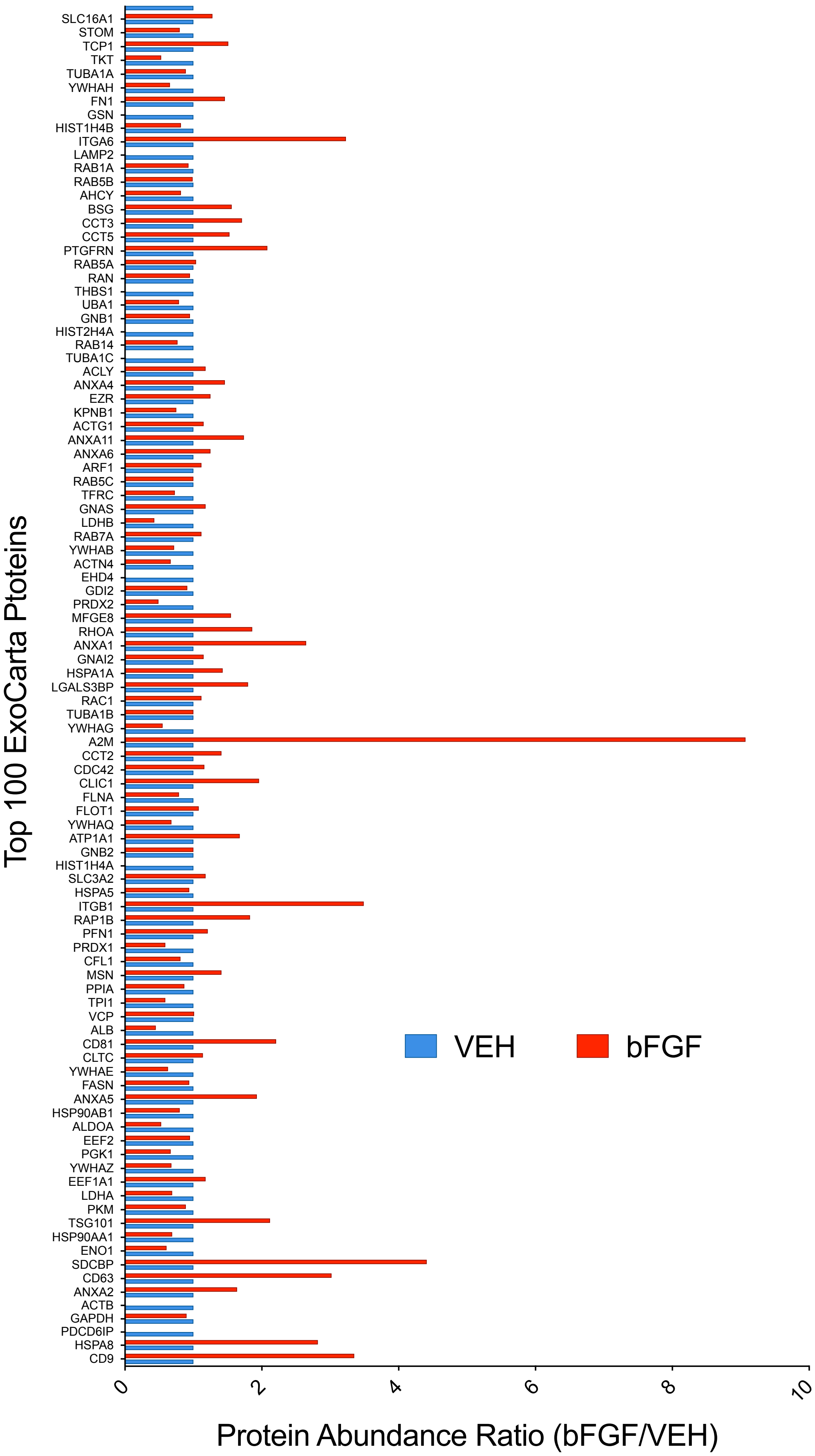
Top 100 ExoCarta Ptoteins
SLC16A1
STOM
TCP1
TKT
TUBA1A
YWHAH
FN1
GSN
HIST1H4B
ITGA6
LAMP2
RAB1A
RAB5B
AHCY
BSG
CCT3
CCT5
PTGFRN
RAB5A
RAN
THBS1
UBA1
GNB1
HIST2H4A
RAB14
TUBA1C
ACLY
ANXA4
EZR
KPNB1
ACTG1
ANXA11
ANXA6
ARF1
RAB5C
TFRC
GNAS
LDHB
RAB7A
YWHAB
ACTN4
EHD4
GDI2
PRDX2
MFGE8
RHOA
ANXA1
GNAI2
HSPA1A
LGALS3BP
RAC1
TUBA1B
YWHAG
A2M
CCT2
CDC42
CLIC1
FLNA
FLOT1
YWHAQ
ATP1A1
GNB2
HIST1H4A
SLC3A2
HSPA5
ITGB1
RAP1B
PFN1
PRDX1
CFL1
MSN
PPIA
TPI1
VCP
ALB
CD81
CLTC
YWHAE
FASN
ANXA5
HSP90AB1
ALDOA
EEF2
PGK1
YWHAZ
EEF1A1
LDHA
PKM
TSG101
HSP90AA1
ENO1
SDCBP
CD63
ANXA2
ACTB
GAPDH
PDCD6IP
HSPA8
CD9
VEH
bFGF
0
2
4
6
8
10
Protein Abundance Ratio (bFGF/VEH)

Supplementary Figure Legends:

Figure S1: CD63-pHluorin is tagged to extracellular vesicles (EVs) and co-localizes with Rab7-positive late endosomes in rat hippocampal neurons. a) Photomicrograph illustrating the lentiviral expression of *p*CD63-pHluorin in rat hippocampal neurons at day 10 post transduction (DPT). Scale bar: 3.5 µm **b**) Immunocytochemical staining (ICC) from CD63-pHluorin-transduced neurons stained with an antibody against Rab7 (red) and counterstained with DAPI (blue). Scale bar: 3.5 µm. **c**) Bar graph showing the Pearson's correlation coefficient (PCC) for CD63-pHlurin with different antigens of the endolysosomal pathway. As expected, the highest PCC was obtained from correlating the pHluorin signal with the signal from a GFP antibody, followed by Rab7. **d**) Photomicrographs demonstrating an increase in GFP-fluorescence in response to perfusion of CD63-pHlurin-transfected neurons with 50 mM NH_4Cl. Scale bar: 5.5 µm. **e**) Graph illustrating the NH_4Cl-evoked pHluorin fluorescence signal as a function of time. **f**) Western blot illustrating the segregation of GFP, CD81 and Alix/AIP1 to different iodixanol densities. Alix/AIP1 and CD81 segregate to densities of 1.10 to 1.12, whereas GFP exhibited a broader distribution, indicating the heterogeneity of GFP-positive extracellular vesicles (EVs). **g-i**) Graphs illustrating the correlation of the signal intensities between different EV markers in each Western blot (n = 48/antibody). For correlation, a linear regression has been conducted and an R-squared calculated.

Figure S2 Thapsigargin has no immediate effect on neuronal EV release. a) Photomicrographs from cultured hippocampal neurons imaged during the application

of thapsigargin (1 μM). Scale bar: 50 μm. **b,c**) Application of thapsigargin (10 μM) has no effect on pHluorin fluorescence. Coloured circles indicate the region of interest (ROIs) from which the signal was analysed and displayed over time. Scale bar: 25 μm.

Figure S3: Treatment with bFGF has no effect on the expression of CD63-pHluorin or on EV release. a) Western blot illustrating the abundance of total pHluorin, biotinylated (= surface) pHluorin and GAPDH. **b,c**) Bar graphs illustrating the total and surface pHluorin in cultured hippocampal neurons treated with bFGF for 3, 12, 24 or 48 hrs. **d**) Representative traces from NTA illustrating the particle number/size in CD63-pHlurin-transfected and untransfected neurons. **e**) Bar graph showing no difference between both conditions ($n = 3$, $p = 0.0713$). Data are shown in mean ± s.e.m. For comparison, a one-way ANOVA was used in b) and c) and a two-tailed unpaired t-test was used in e). **$P < 0.01$.

Figure S4: Treatment with bFGF has no effect on neuronal cell death. a) Bar graph illustrating that treatment with bFGF has no statistical significant effect on the release of lactate dehydrogenase (LDH) from cultured neurons when treated with bFGF for 24 hrs (left; $n = 12$; $p = 0.1553$) or 48 hrs (right; $n = 12$; $p = 0.0718$); positive control with 1% Triton X-100 for 15 min. **b,c**) Bar graph and photomicrograph illustrating a comparable average nuclear diameter in bFGF and VEH-treated cells (samples/condition $n = 11804$ and 8300; $p = 0.1021$) suggesting a comparable number of apoptotic nuclei subsequent to bFGF treatment. Data are shown as mean ± s.e.m. For comparison, a two-tailed unpaired t-test was used.

Figure S5 Treatment with bFGF has no effect on the number or size of late endosomes/MVBs. **a**) Photomicrographs of cultured hippocampal neurons illustrating CD63-pHluorin-labeled endosomes in VEH- and bFGF-treated neurons (first and second row). To quantify their number and size, binary images (third row) were created from each group. Scale bar: 5 µm. **b,c**) Treatment with bFGF has no effect on the size or number of CD63-pHluorin-labeled endosomes (particle number: VEH vs. bFGF cells/condition n = 98 and 102; p = 0.9916; particle area: p = 0.8690). **d**) Representative electron microscope (EM) images from VEH- and bFGF-treated neurons stained with gold-conjugate labelled anti-GFP antibody. Sacle bar: 1 µm. **e,f**) Treatment with bFGF had no effect on the size or number of pHluorin-tagged MVBs (particle number: VEH vs. bFGF cells/condition n = 42 and 43; p = 0.3415; particle area: p = 0.0758). Data are shown as mean ± s.e.m. For comparison, a two-tailed unpaired t-test was used.

Figure S6: Correlation analysis of array replicates of VEH and bFGF treated exosomal pallets.

Figure S7: Knockdown of *Vamp3* in bFGF-treated neurons. **a**) Western blot illustrating a strong reduction of Vamp3 in response to transfection with *Vamp3* siRNAs and treatment with bFGF. **b**) Bar graph illustrating a reduced Vamp3 signal und Vamp3-siRNA transfected cells (n = 12/condition). Data are shown in mean ± s.e.m. For comparison, a two-tailed unpaired t-test was used. ***p < 0.001.

Figure S8: Brain-derived neurotrophic factor (BDNF) and nerve growth factor (NGF) increase neuronals EV release. **a**) Representative traces from NTA illustrating the particle number/size in cultured neurons treated with bFGF (50 ng/ml),

BDNF (25 ng/ml) or NGF (50 ng/ml) for 24 hrs. **b,c**) bFGF, BDNF and NGF increase the number of particles in the cell culture medium from cultured neurons without affecting particle size. (n = 8/condition). Data are shown in mean ± s.e.m. For comparison, a two-tailed, paired t-test was used. ***p < 0.001.

Figure S9: Treatment with bFGF differentially affects the abundance of EV-enriched proteins. **a**) Representative Western blot pictures illustrating the protein abundance of Alix, CD81, CD9 and Flotilin-1 in EV-enriched medium pellets from cultured neurons. The respective treatment conditions are indicated above the Western blot. **b**) Bar graph illustrating the protein abundance of Alix, CD81, CD9 and Flotilin-1 in EV-enriched medium pellets from cultured neurons. Treatment of cultured neurons with bFGF has a differential effect on the abundance of the respective proteins in EV-enriched medium pellets. *Alix:* VEH + CON siRNA vs. bFGF + CON siRNA, n = 15, p < 0,0001; VEH + *Vamp3* siRNA vs. bFGF + *Vamp3* siRNA, n = 15, ****p < 0,0001. *CD81:* VEH + CON siRNA vs. bFGF + CON siRNA, n = 12, ***p = 0,001; VEH + *Vamp3* siRNA vs. bFGF + *Vamp3* siRNA, n = 12, **p = 0,002. *CD9:* VEH + CON siRNA vs. VEH + *Vamp3* siRNA, n = 6, **p = 0,0032; VEH + *Vamp3* siRNA vs. bFGF + *Vamp3* siRNA, n = 6, ****p < 0,0001. Data are shown in mean ± s.e.m. For comparison, a one-way ANOVA with multiple comparisons was used.

Figure S10: bFGF affects the abundance of EV-enriched proteins. Bar graph illustrating the relative abundance of the ExoCarta (http://www.exocarta.org) top 100 EV-enriched proteins from VEH (blue) and bFGF-treated neurons (red) as measured by mass spectrometry. The data are extracted from the mass spectrometry measurement in table S1 with an equal total protein concentration in EV-enriched medium pellets from either treatment condition (VEH vs bFGF). Despite an equal

total protein concentration, the abundance of the listed proteins varies up to ~ 10 fold. Data are shown as mean value relative to the VEH condition (n = 6).

Table S1: bFGF affects the abundance of EV-associated proteins. Table indicating the top 1104 differentially abundant proteins in EV-enriched pellets in response to treatment with bFGF (50 ng ml^{-1} for 24 hrs) including the gene names, log ratios and p-values.

Table S2: Effect of bFGF on the abundance of the ExoCarta Top 100 exosomal marker proteins (http://exocarta.org/exosome_markers_new).
